# Comparative Efficacy of Various Exercise Types on Cancer‐Related Fatigue for Cancer Survivors: A Systematic Review and Network Meta‐Analysis of Randomized Controlled Trials

**DOI:** 10.1002/cam4.70816

**Published:** 2025-03-27

**Authors:** Shichen Zhou, Guang Chen, Xiaoyu Xu, Cheng Zhang, Guoming Chen, Yau‐Tuen Chan, Ya Xuan Sun, Jiayan Zhou, Ning Wang, Yibin Feng

**Affiliations:** ^1^ School of Chinese Medicine Li Ka Shing Faculty of Medicine, the University of Hong Kong Hong Kong SAR P.R. China; ^2^ T.H. Chan School of Public Health, Harvard University Boston Massachusetts USA; ^3^ School of Medicine Stanford University Stanford California USA

**Keywords:** cancer‐related fatigue, mind–body intervention, network meta‐analysis, physical exercise, qigong, systematic review, tai chi, yoga

## Abstract

**Background:**

This study compares the effectiveness of 7 types of guideline‐recommended first‐line exercises for cancer‐related fatigue (CRF).

**Methods:**

A comprehensive search was conducted utilizing public databases, including Medline, Embase, Web of Science, and Cochrane Library. Randomized clinical trials examining the effects of aerobic exercise, resistance exercise, stretching exercise, combined aerobic and resistance exercise, Yoga, Qigong, or Tai Chi on CRF in various cancer types were included. A Bayesian network meta‐analysis was used to synthesize the data. Subgroup analyses and sensitivity analyses were used to detect the effect modifiers and to confirm the robustness, respectively.

**Results:**

A total of 33 clinical trials were included in this analysis. Overall, both resistance (SMD, −1.72; 95% CI, −2.81 to −0.63) and Yoga (SMD, −1.27; 95% CI, −1.38 to −1.16) reduced the fatigue severity significantly better than standard care, but there was no significant decrease for other exercise types. For cancer survivors with an age over 55 years, only Yoga showed statistically significant improvement in CRF (SMD, −1.27; 95% CI, −1.38 to −1.16). For patients with an age less than 55 years, both resistance (SMD, −1.75; 95% CI, −2.91 to −0.58) and Yoga (SMD, −1.66; 95% CI, −2.81 to −0.51) reduced the fatigue severity compared to standard care.

**Conclusion:**

Both resistance exercise and yoga showed significant benefits in alleviating CRF compared to standard care. Yoga was particularly effective for cancer survivors over 55 years of age, while resistance exercise and yoga were comparably effective for those under 55 years.

## 1 Introduction

Cancer‐related fatigue (CRF) is a type of physical, emotional, and cognitive exhaustion or tiredness in cancer patients that is persistent, distressing, and subjective, and cannot be entirely relieved by rest or sleep, with more than 80% of cancer patients experiencing fatigue during their survivorship [1, 2]. CRF is the second most prevalent symptom among the 38 late‐stage cancer‐related symptoms, following pain in cancer patients [3]. Two recent systematic reviews and meta‐analyses reported CRF overall prevalence of 52% and 49%, respectively [4, 5]. In addition, a survey in Taiwan showed that CRF is the most common discomfort in gynecologic cancer—a total of 53% were suffering from fatigue and 78% having previously received fatigue‐related management [6].

Various potential pathophysiological mechanisms for CRF have been proposed, including the release of pro‐inflammatory cytokines in the central nervous system [7, 8], hypothalamic–pituitary–adrenal axis (HPA) disturbance leading to dysregulated cortisol release and circadian rhythm disruption [9, 10, 11], and immunity and latent viral reactivation [12, 13]. Poor performance status, chemo‐radiotherapy, sleep disturbance, pain, neuroticism, and depression were found to be associated with the incidence of CRF [5]. Notably, physical inactivity and elevated body mass index (BMI) were also risk factors for CRF. A longitudinal study identified BMI as one of the main predictors of fatigue 6‐ and 42‐months posttreatment among early‐stage breast cancer patients [14].

Although the most updated 2024 American Society of Clinical Oncology/Society of Integrative Oncology (ASCO/SIO) clinical practice guideline for CRF strongly recommended the use of cognitive behavioral therapy and mindfulness‐based programs [15], the overall quality of evidence supporting these two approaches remains moderate to low. The potential treatment approaches for CRF include physical activity interventions, mind–body interventions, psychosocial interventions, and pharmacological approaches based on more than 170 published interventional studies [15]. Regarding pharmacological therapies, a meta‐analysis of 27 eligible randomized controlled trials, including treatments of hematopoietic growth factors, progesterone steroids, the antidepressant paroxetine, and the psychostimulant methylphenidate, suggested that hematopoietic drugs may alleviate CRF related to chemotherapy‐induced anemia [16]. Despite interest in psychostimulants such as methylphenidate, the evidence regarding their efficacy is mixed. Consequently, recent guidelines do not recommend their use in managing fatigue in patients following active treatment [17].

However, cancer survivors evolve CRF in a complex manner. CRF can be (1) initiated by cancer itself due to increased consumption of calories and nutrients burned by cancer cells; (2) induced by cancer treatments, including higher consumption of energy after cancer surgery, irreversible destruction of normal cells during chemotherapy or radiation therapy, and unbalanced metabolism after hormone therapy or immunotherapy; (3) a consequence of side effects of cancer treatment such as anemia, pain, sleep disorders, appetite loss, and diarrhea; (4) interacted with the emotional impact of cancer, including depression, anxiety, and fearfulness. The pathophysiological complexity of CRF is calling for interventions with multiple targets and mechanisms. Therefore, a cost‐effective, tolerable side effect, nonpharmaceutical therapy can be life‐changing for cancer survivors suffering from CRF.

Multiple studies have demonstrated that exercise interventions [18, 19, 20], psychosocial interventions [21, 22] and mind–body interventions, including Yoga, Tai Chi, and Qigong, are effective in reducing fatigue during and after cancer treatment. Moreover, exercise modalities have been extensively investigated for their beneficial effects on cardiometabolic health indices, particularly in individuals with obesity, with increasing evidence supporting their efficacy in improving psychophysiological outcomes [23, 24, 25]. Although the benefits of various exercise programs are well documented and widely recognized [26], a comprehensive summary comparing their relative effectiveness in mitigating CRF remains largely unclear. A network meta‐analysis is particularly valuable in advancing this field, as it integrates both direct and indirect evidence, allowing for a systematic comparison of diverse interventions and their respective efficacies. Therefore, we conducted a network meta‐analysis not only to combine direct and indirect evidence [27] regarding exercise interventions for CRF but also to perform subanalyses as a secondary aim to further clarify the most effective modalities and patient‐specific considerations.

The aim of this systematic review and network meta‐analysis was to compare the effects of physical activity and mind–body interventions on CRF. Additionally, we conducted subgroup analyses as a secondary aim to identify potential effect modifiers of exercise interventions on CRF, including age.

## 2 Materials and Methods

This systematic review, including a Preferred Reporting Items for Systematic Reviews and Meta‐Analyses for Network Meta‐Analyses (PRISMA‐NMA), followed the protocol that was prospectively registered in PROSPERO (the International Prospective Register of Systematic Reviews, registration no. CRD42024627952). The systematic review procedures in this study adhered to the guidelines established by the Cochrane Back Review Group [28] and the implementation of PRISMA in the fields of sports, rehabilitation, exercise medicine, and sports science [29]. The results of the network meta‐analysis were reported in accordance with the extended guidelines of the PRISMA‐NMA [30]. In this study, we used a Bayesian network meta‐analysis approach, as it provides a more robust method for comparing treatment effects [31].

### 2.1 Literature Search Strategy and Study Selection

Potential literature was comprehensively searched using four electronic databases: PubMed, EMBASE, Web of Science, and The Cochrane Library, from their inception to April 2024. The data retrieval strategy was based on the PICOS framework [32]: (P) Population: patients diagnosed with any type of cancer; (I) Intervention: any type of exercises, Qigong, Tai Chi, or Yoga; (C) Control Group: any type of exercises, Qigong, Tai Chi, Yoga, standard care, or health education; (O) Outcomes: any assessment of CRF symptoms; (S) Study Type: randomized controlled trials (RCT). Subsequently, the references within the pertinent literature were meticulously screened to ascertain additional relevant articles. The detailed literature search strategy is delineated in Table S1.

Covidence platform was used for literature selection. After uploading all literature to Covidence, two researchers independently screened the titles and abstracts of all potential literature after comprehensive searching. Subsequently, the abstracts of the remaining literature were reviewed to determine their eligibility for exclusion. Then, two researchers independently read the full‐text articles using the inclusion and exclusion criteria and assessed whether the studies were included. Duplicate titles, conference papers, protocols without results, retrospective studies, nonrandomized controlled trials, and pilot studies were excluded. During the selection process, the Covidence platform automatically cross‐checked and compared the judgments of the two researchers for each potential literature. In case of any disagreement, a third reviewer was consulted for discussion and resolution.

### 2.2 Inclusion and Exclusion Criteria

Studies were eligible for inclusion if they met the following criteria: (1) the study population was any type of cancer; (2) any type of exercise training (aerobic exercise, resistance exercise, and combined training), Qigong, Yoga, or Tai Chi as interventions; (3) the control group was any type of exercise training, or standard and routine care, including usual care, waitlist control, and education; (4) outcome measures utilized one or more of the following assessment tools: Brief Fatigue Inventory (BFI), Stimulus to Fatigue Ratio (SFR), Fatigue Symptom Inventory (FSI), Functional Assessment of Chronic Illness Therapy–Fatigue (FACIT‐F), Fatigue Symptom Inventory‐Short Form (MFSI‐SF), Piper Fatigue Scale (PFS), or European Organization for Research and Treatment of Cancer Quality of Life Questionnaire‐Lung Cancer 13 (EORTC QLQ‐LC13); and (5) the study design was a randomized clinical trial (RCT).

The exclusion criteria were: (1) nonrandomized controlled trials, including quasi‐randomized controlled trials; (2) animal tests; (3) studies that did not report specific outcome measures that can be synthesized; (4) conference abstracts, case reports, and pilot studies; and (5) publications not in the English language.

### 2.3 Data Extraction, Risk of Bias Assessment, and Certainty of Evidence

We extracted detailed publication information (study ID, first author, and year of publication), demographic information (age, sex, body weight, and body height), clinical characteristics (cancer type, cancer stage, and treatment approaches), clinical trial design, study sample size, intervention procedures (frequency, intensity, and duration of exercise intervention) and outcomes (fatigue outcome measurements) from the studies using a standardized spreadsheet on the Covidence platform. For studies that did not report the standard deviation of the change in primary outcome measures, the standard error of the change was calculated assuming a zero correlation between baseline and postintervention measures, using the following formula: [33]

$$\sqrt{{\left(\frac{\mathrm{SD}}{n_{\text{baseline}}}\right)}^{2}+{\left(\frac{\mathrm{SD}}{n_{\text{post}}}\right)}^{2}}$$

The risk of bias was assessed for each included study by two researchers independently based on the Cochrane Risk of Bias tool version 2 (RoB 2) [34]. This tool evaluates the risk of bias in the following aspects: selection bias and confounding factors induced by inappropriate randomization, performance bias induced by inappropriate blinding, attrition bias induced by missing data, detection bias induced by inconsistent measurements of outcomes, and reporting bias induced by selection of reported results. According to the criteria above, the trials were classified as having low, high, or unclear risk of bias. The certainty of the evidence for the ranking of network interventions was assessed using the Grading of Recommendations for Assessment, Development, and Evaluation (GRADE) of Network Meta‐Analysis approach. The certainty of the evidence was independently graded by two investigators. In cases of disagreement, the final decision was made by the research team after consultation.

### 2.4 Data Synthesis and Statistical Analysis

For network meta‐analysis (NMA), Stata (Version 16.0) was used to generate a network diagram to illustrate the direct and indirect comparative relationships between the interventions included in the trials [35]. All outcome measures from various physical activity interventions were treated as continuous variables and presented as mean, standard deviation (SD), and mean difference (MD, representing the absolute difference between treatment and control groups, calculated using the same sample size) or standardized mean difference (SMD, representing the group mean divided by the standard deviation between participants, suitable for analyzing data from trials of different sizes), with 95% confidence intervals (CI) [36]. Given the use of multiple fatigue measurement tools, we primarily relied on the standardized mean difference (SMD) to ensure outcomes were placed on a comparable scale. Scores were standardized so that higher values consistently indicated greater fatigue, thereby facilitating combined synthesis across different instruments.

Network meta‐analysis (NMA) summary and analysis were performed using the “gemtc” package in R software (Version 4.3.2) [37, 38], and Bayesian network meta‐analysis was used to perform pairwise comparisons between the intervention and control groups based on the Markov chain Monte Carlo simulation technique. In addition, considering the heterogeneity between studies, we chose a random effects model for analysis instead of a fixed effects model [39]. The $I^{2}$ statistic was applied in a visualized forest plot to assess the heterogeneity between studies, and $I^{2}$ values < 25%, 25%–50%, and > 50% were considered to indicate low, moderate, and high heterogeneity, respectively [39]. To assess consistency, we evaluated the inconsistency of each network using a random effects design‐treatment interaction model (Direct and indirect estimates are statistically similar).

In a Bayesian framework, network meta‐analysis (NMA) estimates the overall ranking of interventions by calculating the surface under the cumulative ranking probability curve (surface under the cumulative ranking, SUCRA) for different interventions. The larger the SUCRA value, the greater the probability of being the most effective intervention, which is equal to 1 when the treatment is determined to be the best and 0 when the intervention is determined to be the worst [40]. In determining the ranking of SUCRA, it is important not only to compare the areas under the cumulative ranking probability curves of different physical activity interventions but also to carefully interpret the clinical significance of these interventions.

The criteria for subgroup analyses were guided by clinical and practical considerations, particularly age, as it can influence exercise tolerance and response. In this dataset, the median age across all included trials was approximately 55 years, which served as the cut‐off to define two subgroups: participants younger than 55 years and participants 55 years and older. Although this threshold is partly arbitrary, it aligns with a commonly observed bifurcation in physiological capacity and comorbidity risk [41], and thus was deemed suitable for exploring potential differences in intervention effects. Our underlying assumption was that older cancer survivors may benefit from different types or intensities of exercise than their younger counterparts, partly due to the lower exercise tolerability associated with advanced age.

To evaluate the stability and reliability of our findings, we performed a sensitivity analysis by excluding studies deemed high risk of bias based on the RoB 2 framework, as well as those with sample sizes smaller than the median (*n* = 20). We then conducted the network meta‐analysis to evaluate whether these lower‐quality articles influenced the overall effect size. In addition, we examined potential effect modifiers in further subgroup analyses of primary outcomes, including the median age (under 55 years and over 55 years).

## 3 Results

### 3.1 Characteristics of Included Studies

**FIGURE 1:**
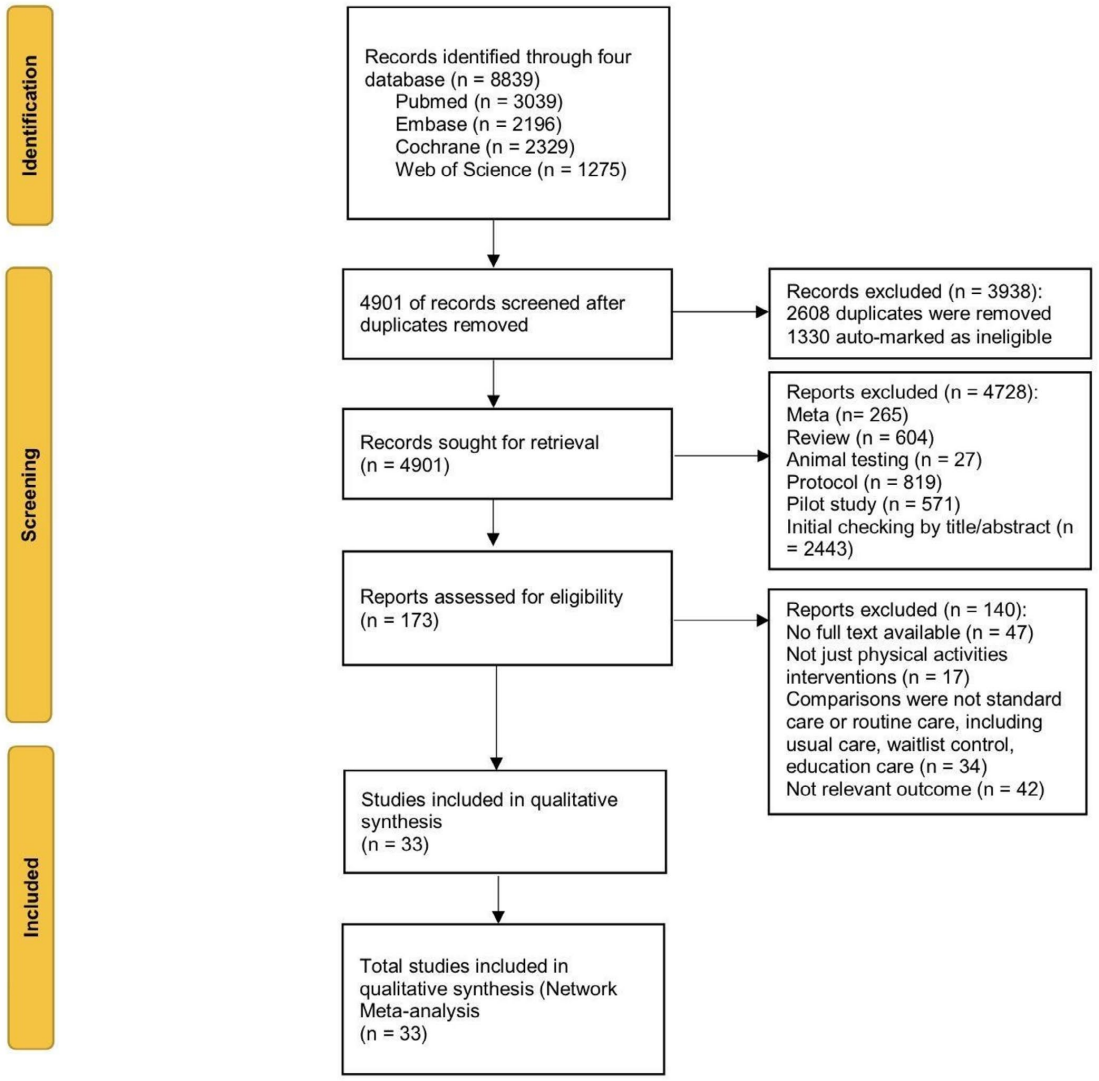
Flow chart of the process of study selection.

**FIGURE 2:**
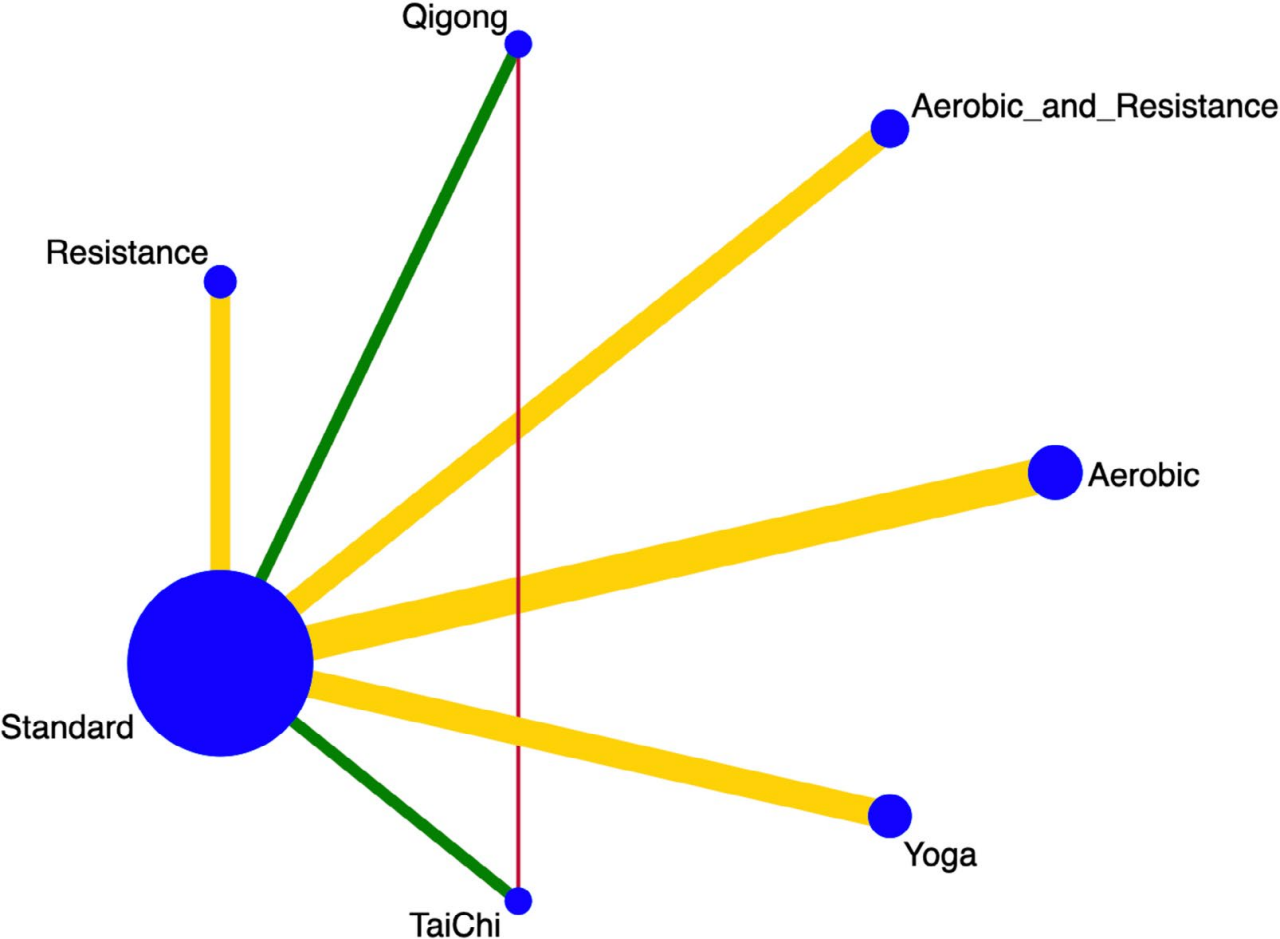
Network plot of available direct comparisons.

After a comprehensive literature search in multiple databases, we initially identified 4901 articles. The team assessed titles and abstracts for eligibility and subsequently excluded 4728 articles for various reasons. We evaluated the full texts of 173 articles and ultimately included 33 randomized clinical trials in this systematic review and network meta‐analysis (Figure 1). The characteristics of these studies are summarized in Table 1. Among the 33 trials, the mean age of the participants ranged from 18 to 71 years, and the mean baseline BMI varied from 23 to 30. Cancer types included head and neck, lung, breast, gynecological, colorectal, testicular, prostate, and lymphoma. The sample sizes in the included studies ranged from 20 to 358. Outcome assessment tools included BFI, SFR, FSI, FACIT‐F, MFSI‐SF, PFS, EORTC, and QLQ‐LC13. Figure 2 illustrates the network diagram depicting the effects of seven exercise types on CRF.

**TABLE 1:** Baseline characteristics of studies included in the network meta‐analysis focusing on patients with cancer‐related fatigue (CRF).

Study (first author, year, country)	Sample size at baseline	Mean age ± SD (years)	BMI (kg/m^2^)	Cancer type and cancer stage	Intervention: Type of exercise program; frequency; length of exercise; duration	Control group	Outcomes
B.R.Villumsen, 2019, Denmark	46	I: 53 ± 11 C: 51 ± 11	I: 29.8 ± 0.6 C: 29.1 ± 0.7	Breast Cancer (I–III)	Aerobic; 4 times per week; 90 min; 10‐week	Standard care	FACT‐F
C.C.Henke, 2014, Germany	43	Older than 18 years	Not reported	Lung Cancer (III–IV)	Resistance; 5 days a week; NA; 64‐week	Standard care	Fatigue_QLQ_C30
C.J.Taso, 2014, Taiwan	60	Total: 49.27 ± 10.23	Not reported	Breast Cancer (I–III)	Yoga; 2 times per week; 60 min; 8‐week	Standard care	BFI
C.L.Hwang, 2012, Taiwan	24	I: 61 ± 6.3 C: 58.5 ± 8.2	I: 23.1 ± 2.6 C: 22.6 ± 2.4	Lung Cancer (III–IV)	Aerobic; 3 times per week; 30‐40 min; 8‐week	Standard care	Fatigue_QLQ_LC13
C.M. Donnelly, 2011, United Kingdom	33	I: 53.5 ± 8.7 C: 52.1 ± 11.8	I: 29.6 ± 8.3 C: 30.0 ± 7.8	Gynecological Cancer (I–III)	Mixed; 5 days per week; 30 min; 12‐week	Standard care	MFSI‐SF
D.Reis, 2013, United States	41	I: 54 ± 11.1 C: 59 ± 10.7	Not reported	Breast Cancer (I–III)	Aerobic; 3 times per week; 20‐60 min; 12‐week	Standard care	PFS‐R
F.Shobeiri, 2016, Iran	60	I: 42.7 ± 9.6 C: 43.5 ± 8.6	I: 27.68 ± 4.90 C: 27.21 ± 4.40	Breast Cancer (I–II)	Aerobic; 2 times per week; 40‐60 min; 10‐week	Standard care	Fatigue_QLQ_C30
J.M.Broderick, 2013, Ireland	43	I: 52.3 ± 8.3 C: 51.2 ± 10.3	I: 26.6 ± 4.0 C: 26.7 ± 3.6	De‐conditioned Cancer (I–III)	Aerobic; 2 times per week; 45 min; 8‐week	Standard care	FACIT‐F
J.Y.Kim, 2019, Republic of Korea	71	Total: 56.8 ± 10.2	I: 23.7 ± 2.9 C: 23.3 ± 3.6	Colorectal cancer (II–III)	Mixed; the first 6 weeks: 18 h per week/after 6 weeks 27 h per week; 12‐week	Standard care	FACIT‐F
K.A.Nyrop, 2017, United States	78	Age 21 or older	Total: ≥ 30 kg/m^2^	Breast Cancer (I–IV)	Aerobic; at least 150 min per week; NA; 6‐week	Standard care	Fatigue, VAS
K.Gokal, 2016, United Kingdom	50	I: 52.08 ± 11.7 C: 52.36 ± 8.9	I: 27.20 ± 4.82 C: 28.25 ± 5.83	Breast Cancer (I–III)	Aerobic; 5 times per week; 30 min; 12‐week	Standard care	FACT‐F
K.S.Courneya, 2003, Ireland	53	I: 59 ± 5 C: 58 ± 6	Not reported	Breast Cancer (I–III)	Resistance; 3 times per week; 15‐35 min; 15‐week	Standard care	FACT‐FS
K.S.Courneya‐AET, 2007, Ireland	158	Mean 49 years	Not reported	Breast Cancer (I–II)	Aerobic; 3 times per week; 60 min; 24‐week	Standard care	FACT‐F
K.S.Courneya‐RET, 2007, Ireland	162	Mean 49 years	Not reported	Breast Cancer (I–II)	Resistance; 3 times per week; 8–12 repetitions; 24‐week	Standard care	FACT‐F
K.S.Courneya, 2009, Ireland	122	I: 52.8 ± 14.75 C: 53.5 ± 15.5	I: 27.4 ± 4.5 C: 26.7 ± 5.4	Lymphoma (I–IV)	Aerobic; 3 times per week; 15–20 min (first 4 weeks), increased 5 min per week to 40–45 min	Standard care	FACT‐F
M.L.McNeely, 2008, Canada	52	I: 53 ± 11 C: 57 ± 8.25	Not reported	Head and neck cancer (I–IV)	Mixed; a minimum of 2 times per week; NA; 12‐week	Standard care	FACT‐F
S.B.Santagnello, 2020, Brazil	26	I: 52.1 ± 10.1 C: 59 ± 9.2	I: 26.2 ± 3.3 C: 25.5 ± 6.5	Breast Cancer (I–III)	Resistance; 3 times per week; between 8 and 12 repetitions per set; 12‐week	Stretching exercise	SFR
S.C.Adams, 2018, Canada	63	I: 44 ± 11.6 C: 43.3 ± 9.9	Not reported	Testicular cancer (I–IV)	Mixed; 3 times per week; 35 min; 12‐week	Standard care	CRF
S.Cataldi, 2019, Spain	20	I: 53.3 ± 19.2 C: 52 ± 15.7	Not reported	All types of cancer (Not reported)	Resistance; 2 times per week; 35 min; 12‐week	Standard care	SFR
T.R.S.Paulo, 2019, B	36	I: 63.2 ± 7.1 C: 66.6 ± 9.6	I: 28.9 ± 5.2 C: 31.5 ± 6.2	Older breast cancer (I–III)	Mixed; 3 times per week; 40 min; 36‐week	Stretching exercise	Fatigue_QLQ_BR23
W.Ndjavera, 2020, United Kingdom	50	I: 71.4 ± 5.4 C: 72.5 ± 4.2	I: 28.4 ± 3.1 C: 27.7 ± 3.4	Prostate cancer (Not reported)	Mixed; 2 times per week; 60 min; 12‐week	Standard care	FACIT‐F
W.Zhou, 2018, China	114	18 years ≤ age ≤ 70 years	Not reported	Nasopharyngeal Carcinoma (III–IV)	Aerobic; 5 times per week; 60 min; 80‐week	Standard care	MFSI‐SF
Namazinia, 2023, Iran	78	I: 49 ± 9.6 C: 45.2 ± 12.6	Not reported	All types of cancer (Not reported)	Yoga; 4 times per week; 20–30 min; 4‐week	Standard care	Fatigue_QLQ_C30
Lisa K. Sprod, 2023, United States	97	I: 67.91 ± 1.05 C: 64.81 ± 0.59	Not reported	Older cancer (I–IV)	Yoga; 2 times per week; 75 min; 4‐week	Standard care	MFSI‐SF
Miek C. Jon, 2018, Sweden	76	I: 51 ± 8 C: 51 ± 7.3	Not reported	Breast Cancer (I–III)	Yoga; 1 time per week; 75 min; 12‐week	Standard care	MFSI‐SF
Po‐Ju Lin, 2019, United States	358	I: 55 ± 11.1 C: 53.7 ± 9.3	Not reported	All types of cancer (Not reported)	Yoga; 2 times per week; 75 min; 4‐week	Standard care	MFSI‐SF
Kiecolt‐Glaser, 2014, United States	200	I: 51.8 ± 9.8 C: 51.6 ± 9.6	I: 27.9 ± 5.3 C: 27.6 ± 6.0	Breast Cancer (I–III)	Yoga; 2 times per week; 90 min; 12‐week	Standard care	MFSI‐SF
J.E. Bower, 2012, United States	31	I: 54.4 ± 5.7 C: 53.3 ± 4.9	I: 24.0 ± 2.5 C: 25.3 ± 3.4	Breast Cancer (I–III)	Yoga; 2 times per week; 90 min; 12‐week	Standard care	FSI
Li‐Li Zhang, 2016, China	91	Total: 62.8	Not reported	Lung Cancer (I–IV)	Tai Chi; 1 session/2 days (10‐21th day)/CTC; 4 CTCs	Stretching exercise	MFSI‐SF
Aeyong E, 2007, Repulic of Korea	47	Total: 44.2 ± 6.65	Not reported	Breast Cancer (I–II)	Tai Chi; 2 times per week; 60 min; 12‐week	Standard care	SFR
MCQUADE JL, 2017, United States	42	I: 62.2 ± 7.4 C: 65 ± 5.91	Not reported	Prostate cancer (I–III)	Qigong; 3 times per week; 40 min; 6 or 8 weeks	Stretching exercise	BFI
Larkey LK, 2015, United States	45	Total: 58.8 ± 8.94	I: 27.1 ± 5.33 C: 26.6 ± 3.62	Breast Cancer (0–III)	Tai Chi; 5 times per week; 30 min; 12‐week	Qi gong	FSI
B. Oh, 2010, Australia	162	I: 60.1 ± 11.7 C: 59.9 ± 11.3	Not reported	All types of cancer (0–IV)	Qigong; 2 times per week; 90 min; 10‐week	Standard care	FACT‐F
Zhen Chen, 2013, China	123	I: 45.3 ± 6.3 C: 44.7 ± 9.7	Not reported	Breast Cancer (0–III)	Qigong; 5 times per week; 40 min; 5 or 6‐week	Standard care	BFI

Abbreviations: BFI, brief fatigue inventory; EORTC, European Organization for Research and Treatment of Cancer; FACIT‐F, functional assessment of chronic illness therapy–fatigue; FSI, fatigue symptom inventory; MFSI‐SF, fatigue symptom inventory‐short form; PFS, Piper Fatigue Scale; QLQ‐LC13, Quality of Life Questionnaire‐Lung Cancer 13; SFR, Stimulus to Fatigue Ratio.

In this review, we categorized exercise interventions according to the specific activity evaluated in each randomized controlled trial (RCT). We defined “mixed” interventions as those incorporating both aerobic and resistance exercises. Overall, we identified 7 RCTs evaluating Yoga intervention studies, five examining resistance exercise studies, nine assessing aerobic exercise studies, four investigating Qigong studies, four exploring stretching studies, three evaluating Tai Chi studies, and six focusing on combined aerobic and resistance exercise studies. Notably, some RCTs included more than one type of exercise, assigning different interventions to the experimental and control groups within the same trial.

Each node stands for one type of exercise. The size of the nodes is proportional to the number of studies involving the specific exercise type. The color of the line indicates the Risk of Bias of the direct comparison studies (Green for low risk, yellow for some concerns, red for high risk).

### 3.2 Risk of Bias Assessment

**FIGURE 3:**
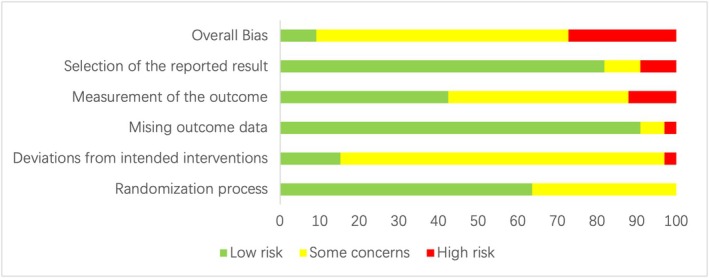
Scale plot of risk of bias for evaluating literature quality evaluation.

Out of the 33 studies we included, 3 studies [42, 43, 44] were classified as low risk, 21 studies [45, 46, 47, 48, 49, 50, 51, 52, 53, 54, 55, 56, 57, 58, 59, 60, 61, 62, 63, 64, 65] were classified as some concerns, and 9 studies [66, 67, 68, 69, 70, 71, 72, 73, 74] were classified as high risk (Figure 3). All included studies demonstrated random allocation, and due to the specific nature of physical activity interventions, blinding of both participants and assessors was challenging, and few studies explicitly blinded participants and staff, as patients and their families were required to provide informed consent before participating in the experiment. In addition, one study [68] had missing results, four studies [66, 67, 69, 70] had unclear reasons for loss to follow‐up, and three studies [71, 72, 73] had incomplete outcome measures. The baseline characteristics of the intervention groups were evenly distributed across all studies, indicating no indications of selective reporting.

### 3.3 Synthesis of Network Meta‐Analysis

**FIGURE 4:**
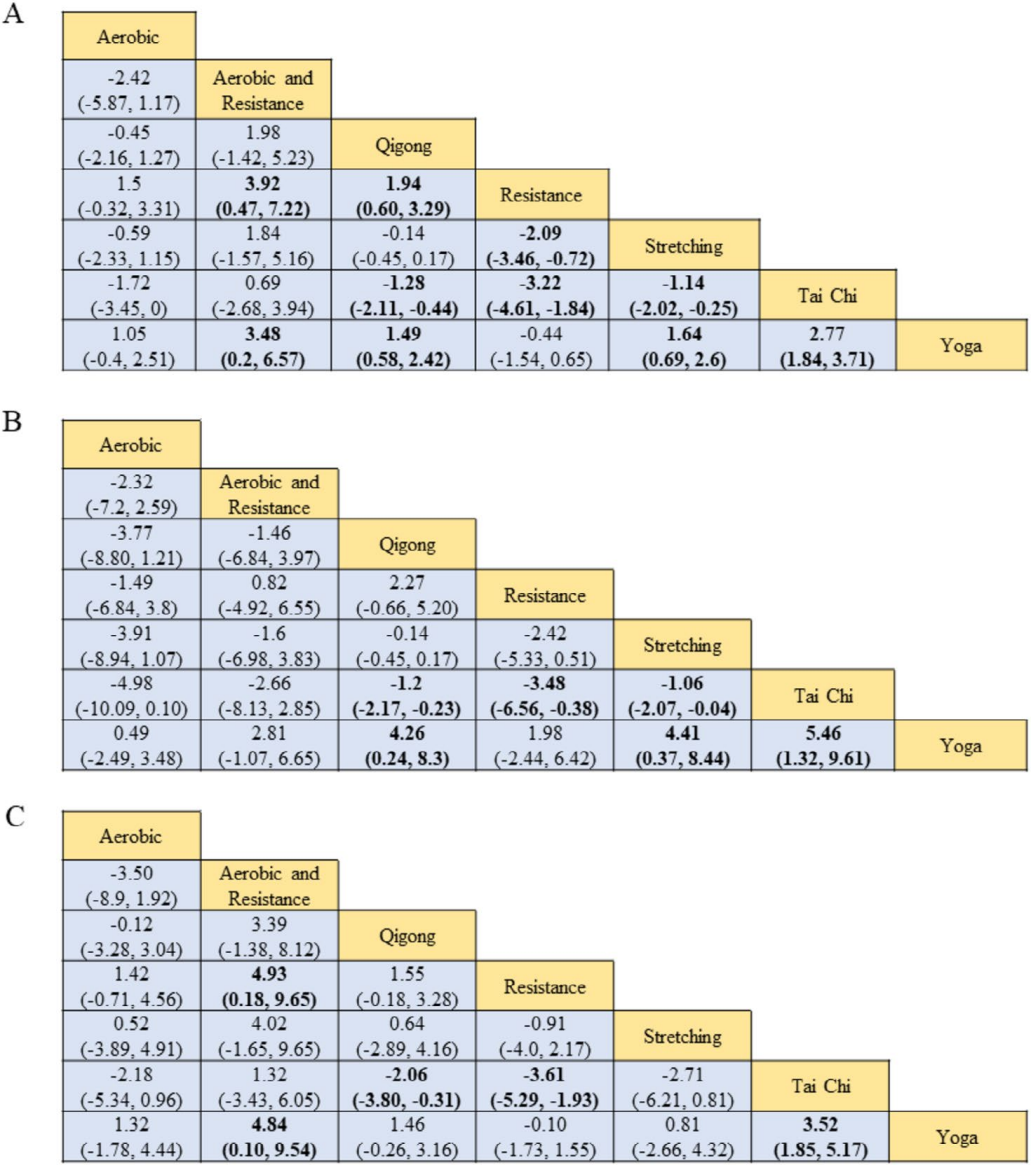
League tables of outcome analyses. (A) Score change in fatigue severity from baseline to the end of exercise intervention. (B) Score change in fatigue severity in subgroup of age over 55 years. (C) Score change in fatigue severity in subgroup of age less than 55 years. The league tables showed the relative effects of each type of exercise (intervention on the column to intervention on the row). The relative effects in this table were estimated by standardized mean difference along with 95% CIs. Bold cells indicated statistical significance (*p* < 0.05). The exercise interventions were listed in alphabetical order.

**FIGURE 5:**
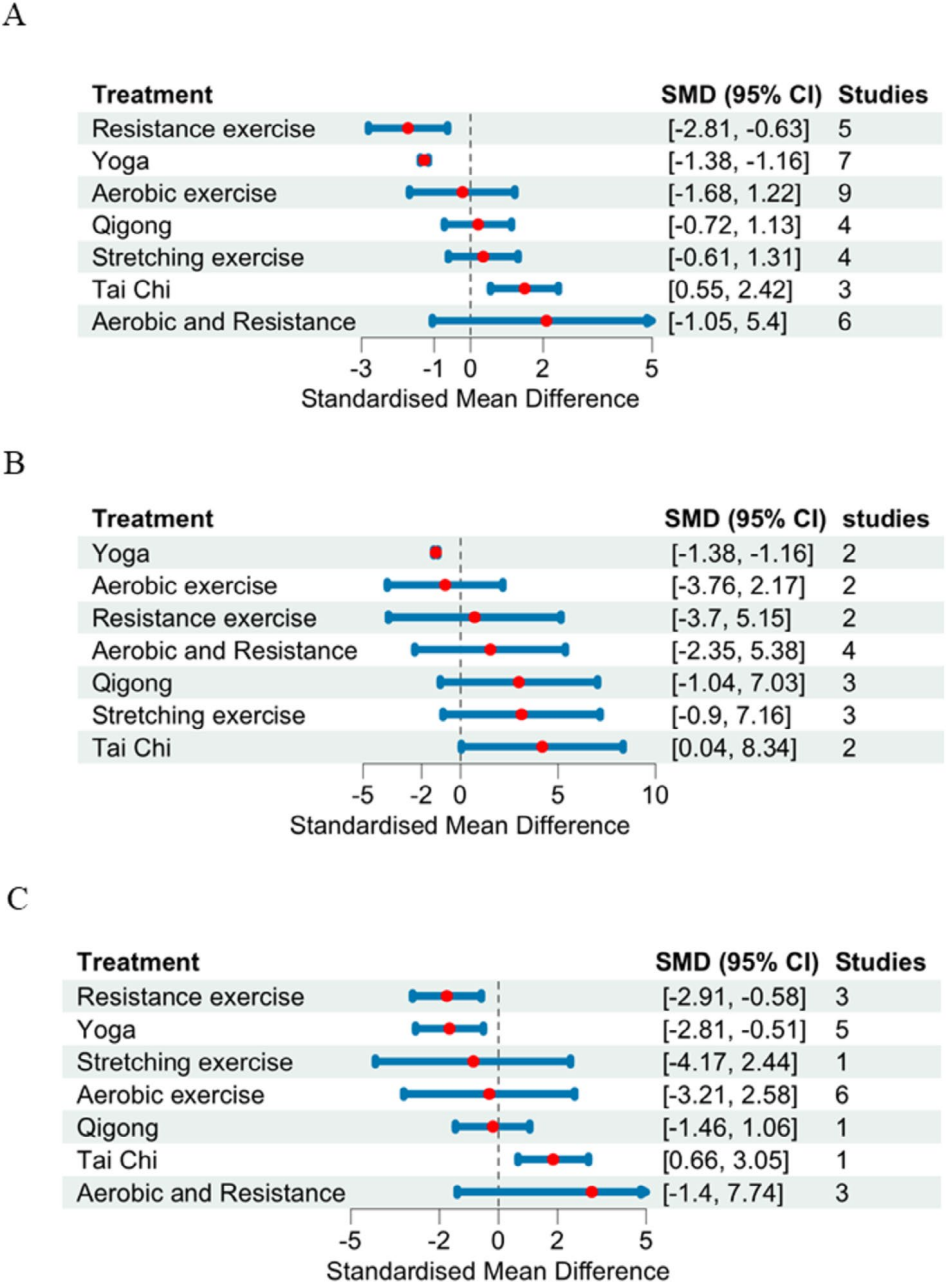
Forest plots of types of exercise on cancer‐related fatigue (CRF). (A) General cancer population. (B) Subpopulation of age over 55 years. (C) Subpopulation of age less than 55 years. The forest plots showed the relative effects of each type of exercise compared with standard care. The relative effects were measured by standardized mean difference with 95% CIs.

Figure 4 shows the league tables for the network meta‐analysis (NMA) estimates of all comparisons between the 7 types of exercises. The summary of findings table and GRADE quality of evidence for each comparison and outcome were presented in the appendix (Table S2). For the outcome of symptom relief of CRF (Figure 5A), both resistance (SMD, −1.72; 95% CI, −2.81 to −0.63) and Yoga (SMD, −1.27; 95% CI, −1.38 to −1.16) reduced the fatigue severity significantly better than standard care, whereas the pooled results did not identify a statistically significant decrease in fatigue severity score by adding aerobic exercise (SMD, −0.23; 95% CI, −1.68 to 1.22), Qigong (SMD, 0.21; 95% CI, −0.72 to 1.13), Tai Chi (SMD,1.49; 95% CI, 0.55 to 2.42), stretching exercise (SMD, 0.35; 95% CI, −0.61 to 1.31), and aerobic and resistance exercise (SMD, 2.18; 95% CI, −1.05 to 5.4) to cancer survivors.

Among physical exercises (Figure 4A), resistance exercise showed better effectiveness than stretching exercise (SMD, −2.09; 95% CI, −3.46 to −0.72), and adding aerobic exercise to resistance exercise did not generate higher improvement compared to resistance exercise alone (SMD, 3.92; 95% CI, 0.47 to 7.22). Among mind–body intervention(Figure 4A), Yoga proved among the best compared to Tai Chi (SMD, −2.77; 95% CI, −3.71 to −1.84) and Qigong (SMD, −1.49; 95% CI, −2.42 to −0.58).

### 3.4 Subgroup Analysis With Stratification for Age

In post hoc analyses of subgroups stratified by age, for cancer survivors over 55 years (Figure 5B), only Yoga, except for all other exercises, showed statistically significant improvement in CRF patients (SMD, −1.27; 95% CI, −1.38 to −1.16). For patients with age less than 55 years (Figure 5C), the network meta‐analyses results showed a similar pattern with the general population—both resistance (SMD, −1.75; 95% CI, −2.91 to −0.58) and Yoga (SMD, −1.66; 95% CI, −2.81 to −0.51) reduced the fatigue severity compared to standard care with a statistically significant difference.

### 3.5 Rank Probabilities

**FIGURE 6:**
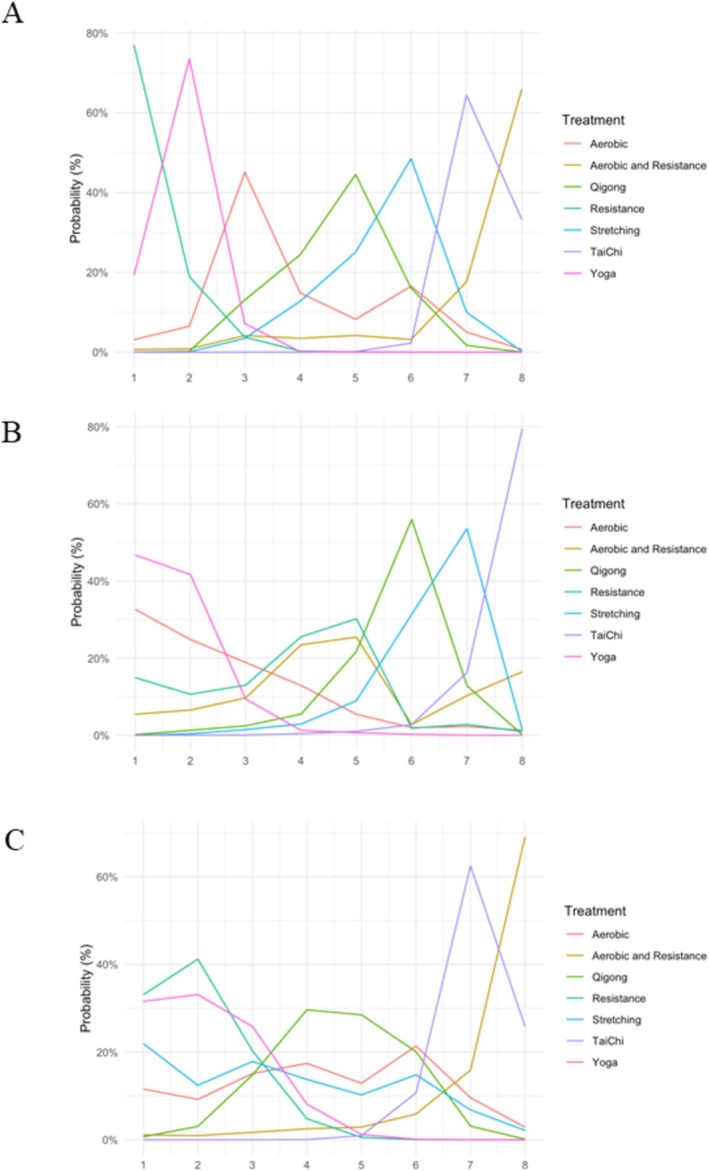
Bayesian ranking profiles of exercise interventions for survivors with cancer‐related fatigue (CRF). (A) General cancer population. (B) Subpopulation of age over 55 years. (C) Subpopulation of age less than 55 years. Profiles indicate the probability of each exercise type being ranked from first to last on CRF relief.

The ranking of exercise types for CRF, as determined by Bayesian network meta‐analysis, was depicted in Figure 6, aligning consistently with the findings of standardized mean differences (SMD) illustrated in Figure 4. For CRF, resistance exercise ranked first with the greatest probability of 77%, followed by Yoga with the likelihood of 74% ranking second. For cancer survivors with age over 55 years, Yoga ranked first with the largest likelihood of 47%, whereas for cancer survivors with age less than 55 years, resistance exercise ranked first with the probability of 33%. This indicated that resistance exercise may be the best option for young cancer survivors, while Yoga may be the best intervention recommendation for older cancer patients with fatigue.

### 3.6 Heterogeneity and Inconsistency Assessment

Figure S1 shows forest plots and heterogeneity estimates for the pairwise comparisons of interventions available. We conducted heterogeneity analyses for CRF within a Bayesian network meta‐analysis framework. Seven studies exhibited minimal (*I* ^2^ = 0%) or low to moderate (*I* ^2^ ≤ 50%) heterogeneity across all comparisons of these different outcomes. High heterogeneity (*I* ^2^ ≥ 50%) was found in comparisons of these outcomes including Yoga versus standard care (98.7%), Qigong versus standard care (61.6%), and Tai Chi versus standard care (71%), stretching versus combined aerobic and resistance exercise (71.3%). In addition, heterogeneity for CRF was 52.4% for standard care various aerobic exercise at baseline.

We assessed the fit of the consistent and inconsistent models using the dispersion information criterion (a Bayesian model evaluation criterion that incorporates fixed effects to gauge model fit and adjusts for complexity) and validated the results using *p*‐values. Our findings indicated that in this network meta‐analysis, the consistent model demonstrated superior fit compared to the inconsistent model (Figure S2), with overall good consistency.

### 3.7 Sensitivity Analysis

The first sensitivity analysis comprised 26 trials involving eight treatments. To mitigate the potential influence of low‐quality studies, reports assessed with a high risk of bias using RoB2 were excluded. This sensitivity analysis was conducted individually for each intervention (Figure S3). Results indicated that resistance exercise and Yoga remained the most effective interventions for improving CRF compared to standard care (Figure S4). In the second sensitivity analysis, which also included 26 trials and eight interventions, studies with small sample sizes (i.e., fewer than 20 participants) were excluded. A network meta‐analysis was then conducted on the remaining trials (Figure S5). The results showed that Yoga was a better treatment than standard care, and other results were not significant (Figure S6).

## 4 Discussion and Conclusion

The main findings of this network meta‐analysis evaluating the comparative efficacy of seven types of exercise for cancer survivors suffering from fatigue were summarized as follows.

1. Both resistance exercise and yoga significantly improved CRF, with resistance exercise showing the most substantial relief in symptom severity among cancer survivors overall.
2. In cancer patients older than 55 years, Yoga proved to be among the most effective in fatigue reduction.
3. Among physical exercises, resistance exercise was better than aerobic exercise, whereas Yoga was better than Qigong and Tai Chi among mind–body interventions.

The comparative effectiveness of resistance exercise over other modalities is supported by its strong performance in sensitivity analyses, but the high effectiveness of yoga, particularly among older patients, highlights its role as a key intervention for CRF management.

The first sensitivity analysis yielded consistent results, indicating that resistance exercise and Yoga remained the most effective interventions for improving CRF compared to standard care, following the exclusion of studies with a high risk of bias. In contrast, the second sensitivity analysis demonstrated that only Yoga showed significant effectiveness over standard care after excluding studies with small sample sizes.

Previous systematic reviews and meta‐analyses [75, 76] showed that physical exercise in general (not specific to one type of exercise) reduced the CRF in breast cancer, prostate cancer, and colorectal cancer. Regarding one specific type of exercise, a systematic review and meta‐analysis [77] with evidence of low certainty showed that progressive resistance exercise was better than routine muscle relaxation care (SMD = −1.11, 95% CI −1.78, −0.43), which was consistent with the findings of our study. Regarding comparisons between different types of exercise, a network meta‐analysis [75] published in 2023 showed that for breast cancer patients, Yoga (SMD = −0.49, 95% CI = −0.75 to −0.22) was the most effective exercise for fatigue relief, followed by aerobic and resistance exercise, which was different from our results. One potential explanation was that the published network meta‐analysis only included breast cancer patients with older ages, while in our study, we found that resistance exercise was the most effective for cancer survivors with age less than 55 years, whereas Yoga was the most effective for older patients. However, a recent systematic review and meta‐analysis [78] reported that high‐intensity interval training significantly reduced both CRF and cancer‐associated pain, drawing from 12 original trials. Based on the findings in our study, low‐ to moderate‐intensity exercise such as Yoga showed better effectiveness in older cancer survivors than high‐intensity exercise such as aerobic exercise, strongly indicating that we should consider whether cancer survivors with fatigue could tolerate the intensity of exercise, namely whether it is feasible for them to receive the exercise training during their survivorship.

Among physical exercises, why did resistance exercise perform better than aerobic exercise for CRF? A recent study illustrated the metabolic profiling change in CRF patients [79], including significantly lower concentrations of tryptophan and valine in CRF patients compared with nonfatigued patients. This study indicated that metabolic change might be the cause of CRF; thus, metabolism could be one of the targets or mediators of CRF treatments. Interestingly, a plasma metabolic profiling study [80] found that resistance exercise was associated with a higher level of metabolites related to the TCA cycle, such as lactate, pyruvate, and malate, while decreased levels of succinate, compared to endurance exercise, which indicated better energy use in resistance exercise. In contrast, endurance exercise increased plasma levels of several of the acyl‐carnitines, indicating accelerated fat metabolism. A few reviews of current evidence [81, 82] also summarized that resistance exercise could alter both lipid, carbohydrate, and amino acid metabolism; thus, increasing anaerobic metabolic capacity and fatigue resistance. In summary, different exercise types produce dissimilar metabolic profiling changes, which partly explain the difference in CRF improvement after varied types of exercise.

Mind–body interventions are a diverse group of therapies that incorporate slow body movement with relaxed breathing, including Tai Chi, Qigong, and Yoga [83, 84]. Compared with physical exercise such as aerobic exercise, mind–body interventions are less intense and often accompanied by meditation along with deep breathing that makes people relaxed, thus intending to affect the physical and mental levels [85]. For older cancer survivors, mind–body interventions might be a better choice due to higher feasibility and compliance. Besides, considering the negative influence from both physical and mental aspects in cancer patients, physical, psychological, and supportive interventions and cares that are beyond survival can be life‐changing [86].

Strengths of this systematic review include the most comprehensive searching, selecting, and synthesizing of evidence to date on all types of exercises for cancer survivors with CRF. To ensure the robustness of the results, both subgroup analyses and sensitivity analyses were conducted within the framework of the research question. Moreover, state‐of‐the‐art methods including the RoB2 assessment tool and the GRADE evaluation system were used to evaluate the risk of bias and provide the quality of evidence, respectively. Limitations of this systematic review include that most of the evidence we summarized in this study was low to moderate, mostly due to the issues of indirectness and imprecision based on GRADE evaluation frameworks. In addition, we lack the pooling of individual cancer patient data, which particularly affected the synthesis of different cancer types, molecular subtypes, and cancer stages. The low statistical power due to fewer studies and smaller sample sizes may have limited the ability to detect significant differences compared to control groups. Future research should prioritize including more robust trials for these modalities to better evaluate their efficacy.

In conclusion, in our network meta‐analysis (NMA), both resistance exercise and Yoga demonstrated greater benefits of CRF relief compared to standard care. Younger cancer survivors (under 55 years) may tolerate and benefit more from higher‐intensity interventions like resistance exercise, whereas older patients (over 55 years) often find low‐ to moderate‐intensity interventions such as yoga more feasible and beneficial. The results of our findings need to be validated in comparative effectiveness randomized clinical trials that directly compare different types of exercise, and long‐term follow‐up is also required to evaluate symptom relapse of CRF.

## Author Contributions

**Shichen Zhou:** conceptualization (equal), data curation (equal), formal analysis (equal), investigation (equal), methodology (equal), resources (equal), software (equal), validation (equal), visualization (equal), writing – original draft (equal), writing – review and editing (equal). **Guang Chen:** conceptualization (lead), investigation (lead), methodology (equal), project administration (equal), resources (lead), software (lead), supervision (equal), validation (equal), visualization (equal), writing – original draft (equal), writing – review and editing (equal). **Xiaoyu Xu:** conceptualization (supporting), formal analysis (supporting), methodology (supporting), software (supporting). **Cheng Zhang:** conceptualization (supporting), funding acquisition (supporting), software (supporting), supervision (supporting). **Guoming Chen:** conceptualization (supporting), data curation (supporting), formal analysis (supporting), resources (supporting), software (supporting). **Yau‐Tuen Chan:** formal analysis (supporting), investigation (supporting). **Ya Xuan Sun:** funding acquisition (supporting), investigation (supporting), resources (supporting). **Jiayan Zhou:** conceptualization (supporting), formal analysis (supporting), funding acquisition (supporting), investigation (supporting), resources (supporting). **Ning Wang:** conceptualization (equal), data curation (supporting), project administration (equal), supervision (equal). **Yibin Feng:** funding acquisition (lead), project administration (lead), supervision (lead), writing – review and editing (lead).

## Conflicts of Interest

The authors declare no conflicts of interest.

## Supporting information


Data S1.

## Data Availability

All data generated or analyzed during this study are included in this published article and its Supporting Information files.
