# Supplementary material for: Comparative Efficacy of Various Exercise Types on Cancer‐Related Fatigue for Cancer Survivors: A Systematic Review and Network Meta‐Analysis of Randomized Controlled Trials
Source: Cancer Med. 2025 Mar 27;14(7):e70816. doi: 10.1002/cam4.70816 (PMC11948276; doi:10.1002/cam4.70816)
Supplement: Supplementary file 1 — Data S1. [file CAM4-14-e70816-s001.docx]

**Supplementary material**

**Table S1.** Searching strategy.

| **Database** | **Search strategy** |
| --- | --- |
| PubMed | (((("Neoplasms"[Mesh]) OR ((((((((((Tumor[Title/Abstract]) OR (Neoplasm[Title/Abstract])) OR (Tumors[Title/Abstract])) OR (Cancer[Title/Abstract])) OR (Cancers[Title/Abstract]))) OR (Malignant Neoplasm[Title/Abstract])) OR (Malignant Neoplasms[Title/Abstract])) OR (Benign Neoplasms[Title/Abstract])) OR (Benign Neopiasm[Title/Abstract]))) AND ("Exercise"[Mesh])) OR ((((((((((((((((((((((Exercises[Title/Abstract]) OR (Physical Activity[Title/Abstract])) OR (Activities, Physical[Title/Abstract])) OR (Physical Activities[Title/Abstract])) OR (Physical Exercise[Title/Abstract])) OR (Physical Exercises[Title/Abstract])) OR (Acute Exercise[Title/Abstract])) OR (Acute Exercises[Title/Abstract])) OR (Tai ji[Title/Abstract])) OR (Exercises,Acute[Title/Abstract])) OR (Tai chi[Title/Abstract])) OR (tai ji quan[Title/Abstract])) OR (Qi Gong[Title/Abstract])) OR (qigong[Title/Abstract])) OR (Exercise, Aerobic[Title/Abstract])) OR (Aerobic Exercise[Title/Abstract])) OR (Aerobic Exercises[Title/Abstract])) OR (Resistance Exercise[Title/Abstract])) OR (Resistance Exercises[Title/Abstract])) OR (Yoga[Title/Abstract])) OR (Exercise Training[Title/Abstract])) OR (yo ga[Title/Abstract]))) AND (((Fatigue[Title/Abstract]) OR (cancer-related fatigue[Title/Abstract])) OR (CRF[Title/Abstract])) AND (((Randomized controlled trial[Title/Abstract]) OR (RCT[Title/Abstract])) OR (Random*[Title/Abstract])) |
| Embase | (Neoplasms or Tumor or Neoplasm or Tumors or Cancer or Cancers or Malignant Neoplasm or Malignant Neoplasms) and (Exercise or Exercises or Physical Activity or activities or Activities, Physical or Acute Exercise or Acute Exercises or Resistance exercise or Resistance exercises or Aerobic Exercise or Aerobic Exercises or Tai ji or tai ji or taichi or tai chi or taijiquan or tai ji quan or Qi Gong or qigong or Yoga or yoga) and (Fatigue or fatigue or cancer-related fatigue or CFR) and (RCT or Randomized controlled trial or Random) |
| Cochrane Central Register of Controlled trials (CENTRAL) | **((Neoplasms or Tumor or Neoplasm or Tumors or Cancer or Cancers or (Neoplasm or Tumor or Tumors or Cancer or Cancers or Malignant Neoplasm or Malignant Neoplasms):ti,ab,kw AND (Exercise or Exercises or Physical Activity or Physical Activities or Acute Exercise or Acute Exercises or Resistance exercise or Resistance exercises or Aerobic Exercise or Aerobic Exercises or tai ji or tai chi or tai ji quan or Qi Gong or qigong or Yoga):ti,ab,kw AND (Fatigue or cancer-related fatigue or CRF):ti,ab,kw AND (Randomized controlled trial or RCT or Random*):ti.ab.kw** |
| Web of science | (TS=(Neoplasms) OR AB=(Tumor OR Neoplasm OR Tumors OR Neoplasia OR Neoplasias OR Cancer OR Cancers OR Malignant Neoplasm OR Malignant Neoplasms OR Benign Neoplasms OR Benign Neoplasm)) AND (TS=(Exercise) OR AB=(Exercises OR Physical Activity OR Activities, Physical OR Activity, Physical OR Physical Activities OR Exercise, Physical OR Exercises, Physical OR Physical Exercise OR Physical Exercises OR Acute Exercise OR Acute Exercises OR Exercise, Acute OR Exercises, Acute OR Exercise, Isometric OR Exercises, Isometric OR Isometric Exercises OR Exercise, Aerobic OR Aerobic Exercise OR Aerobic Exercises OR Exercises, Aerobic OR Exercise Training OR Physical Training OR Resistance exercise OR Resistance exercises OR Taiji OR tai ji OR tai chi OR tai ji quan OR Yoga OR yo ga OR Qi Gong OR qi gong)) AND (AB=(Fatigue OR fatigue OR cancer-related fatigue OR CRF)) AND( AB=(RCT OR Randomized controlled trial OR Random*)) |

**Figure S1.** Forest plots of heterogeneity

**
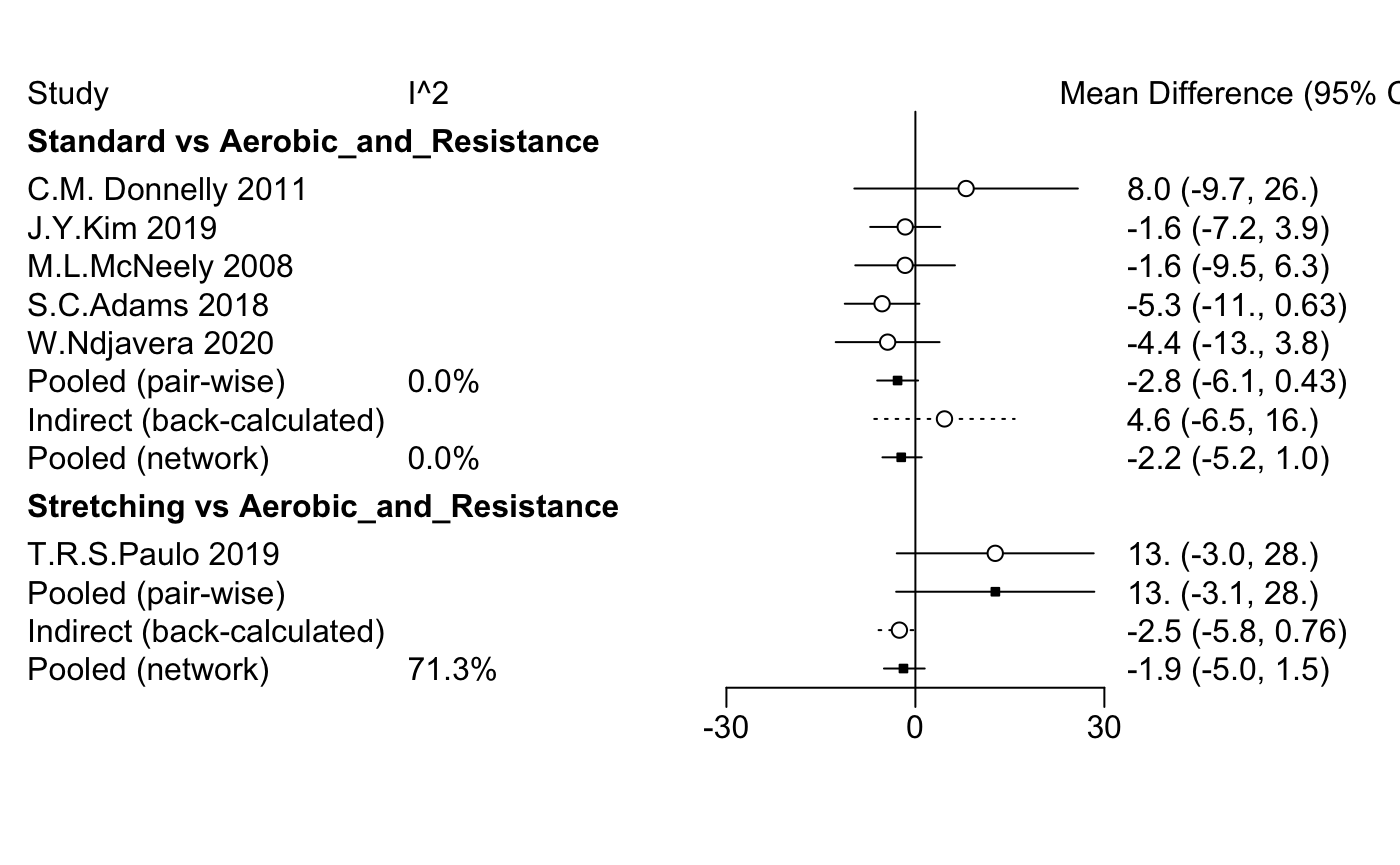
** **
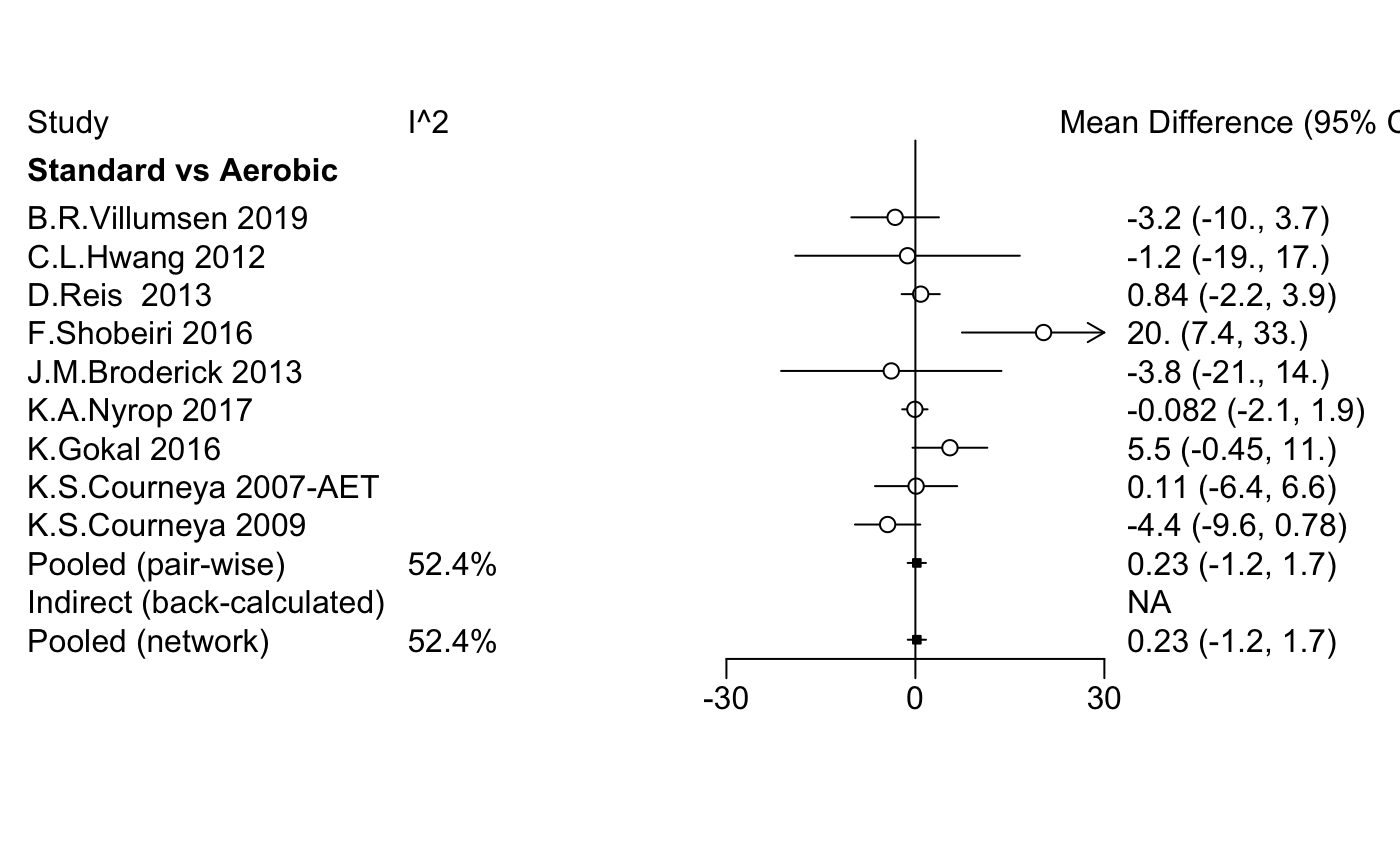
**

**
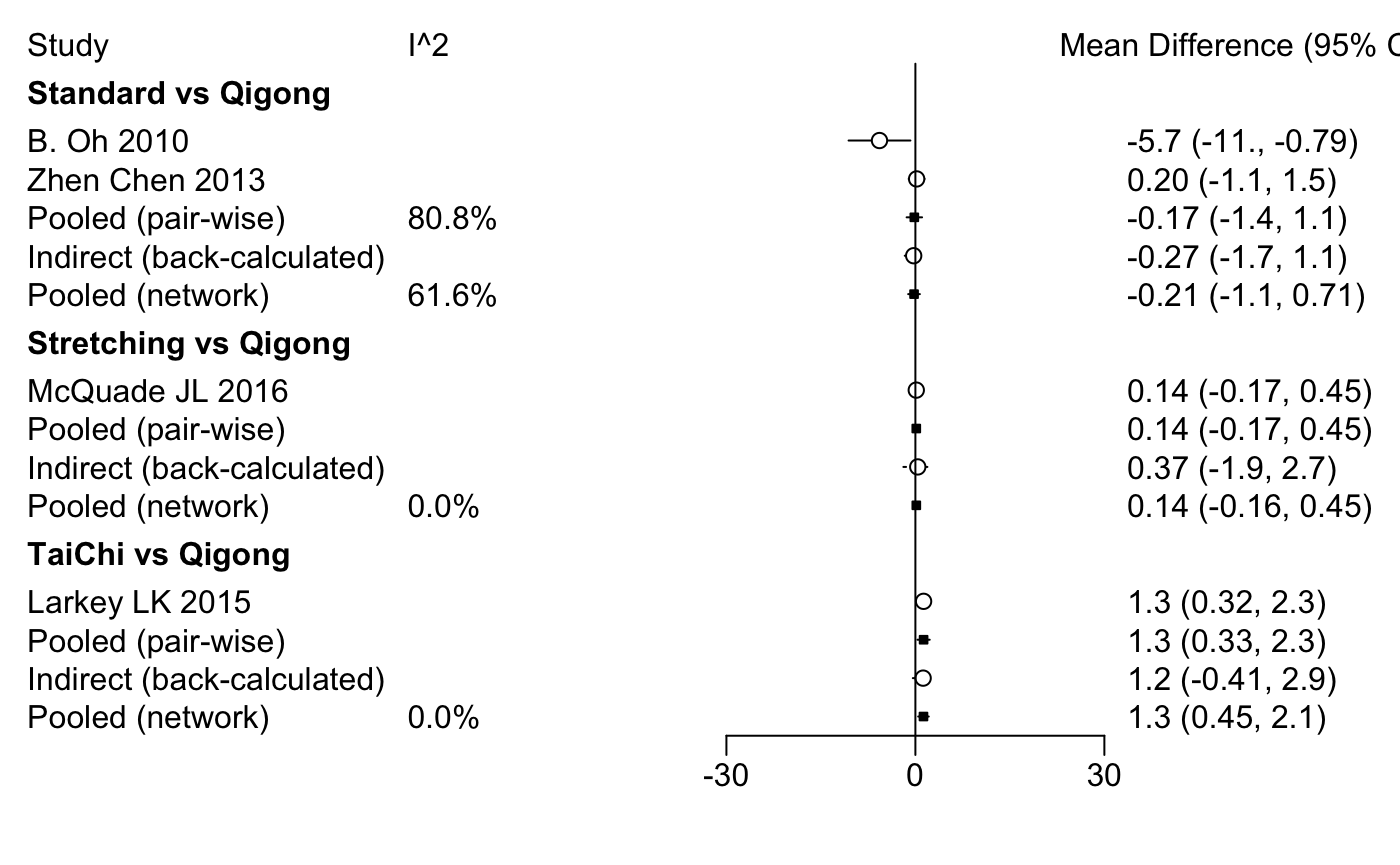
** **
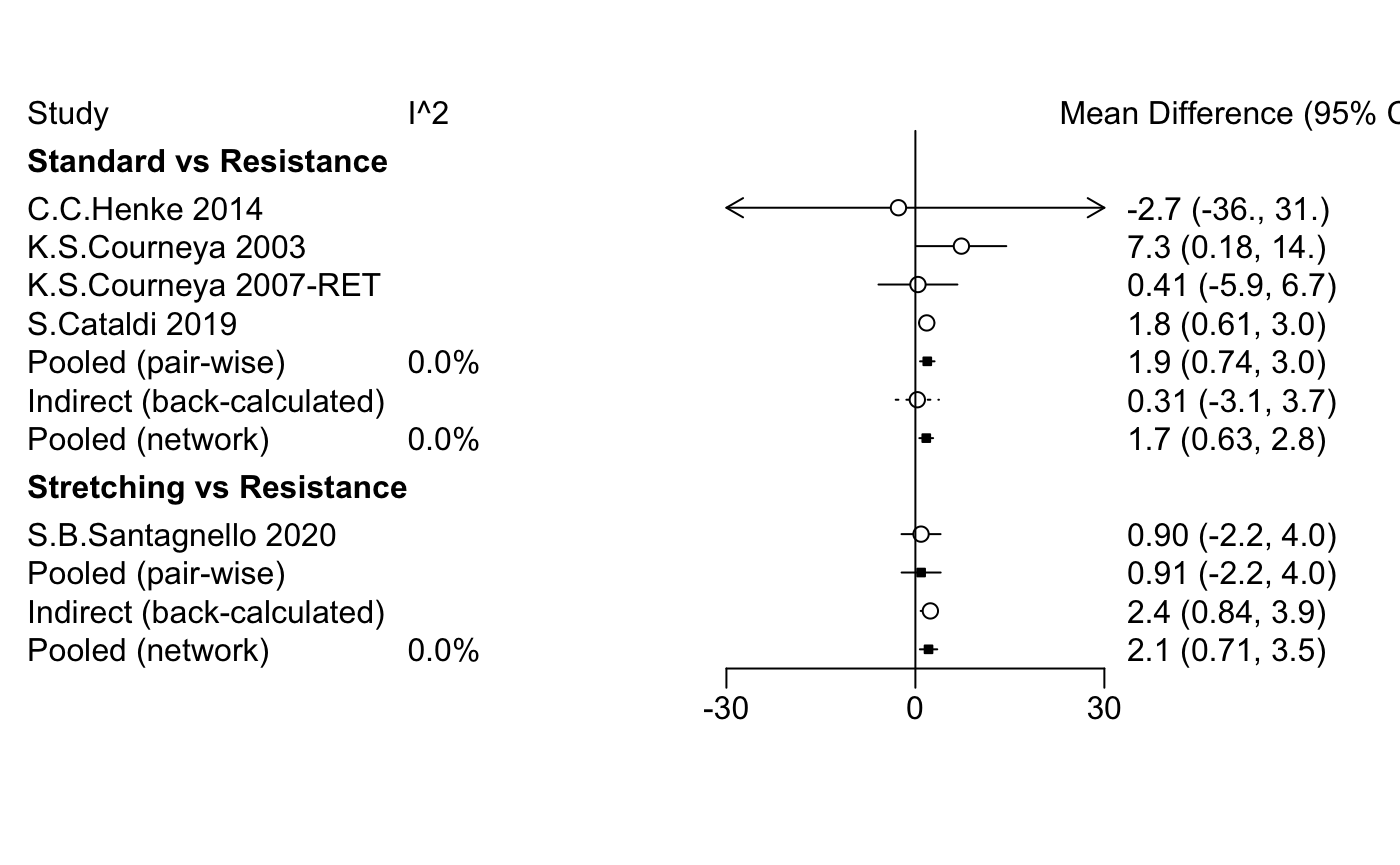
**
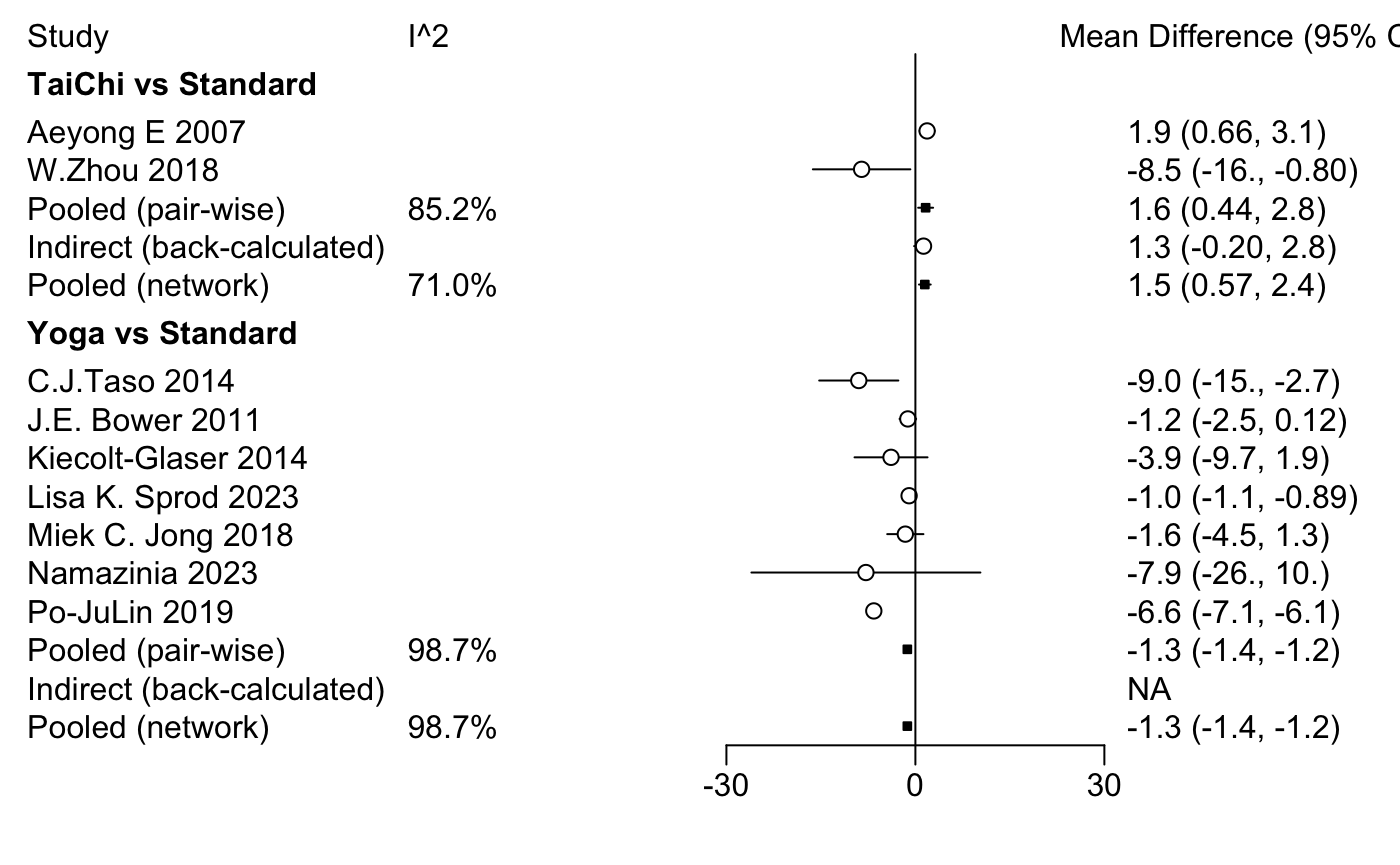

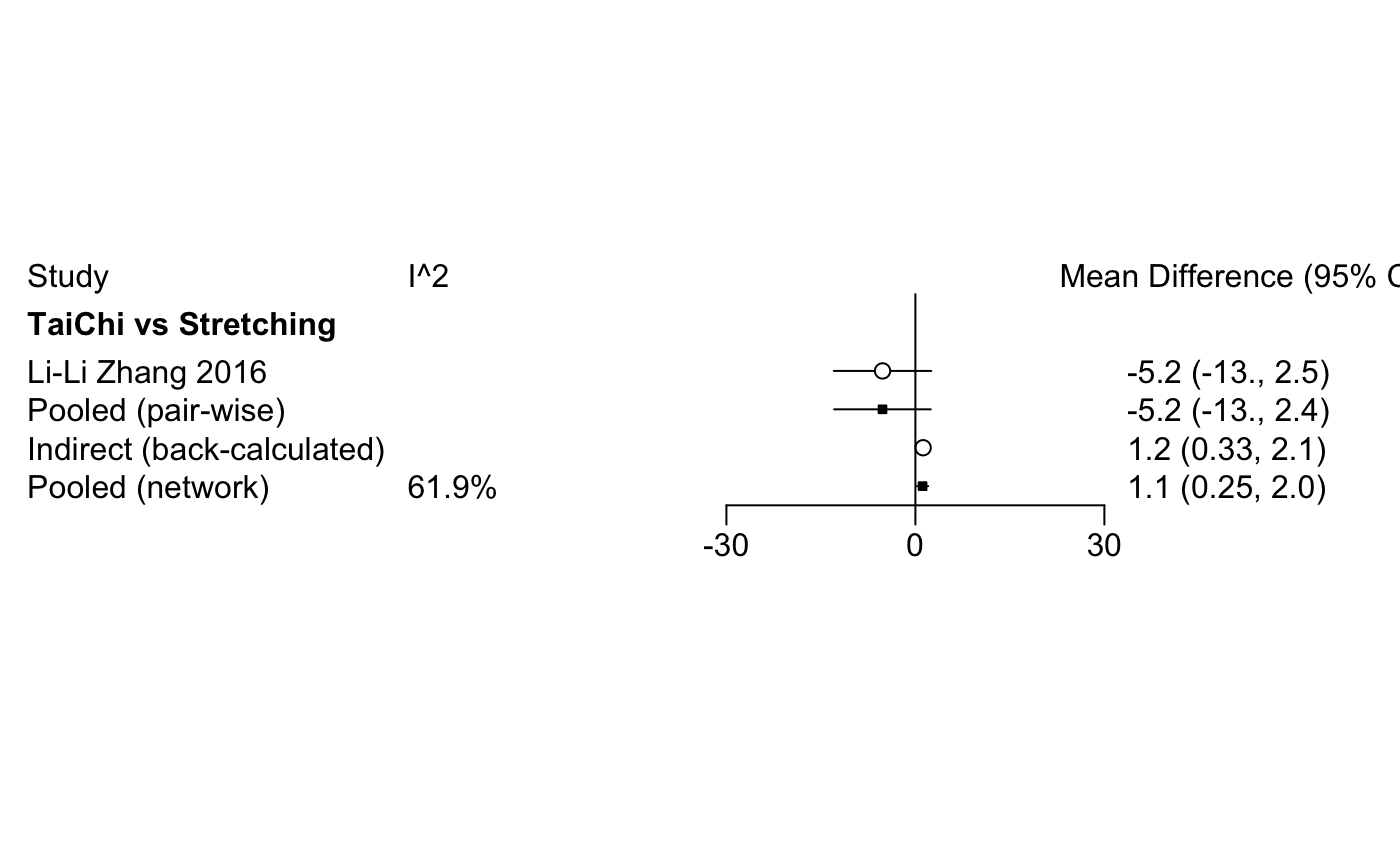


**Figure S2. Forest plot of inconsistency model**

**
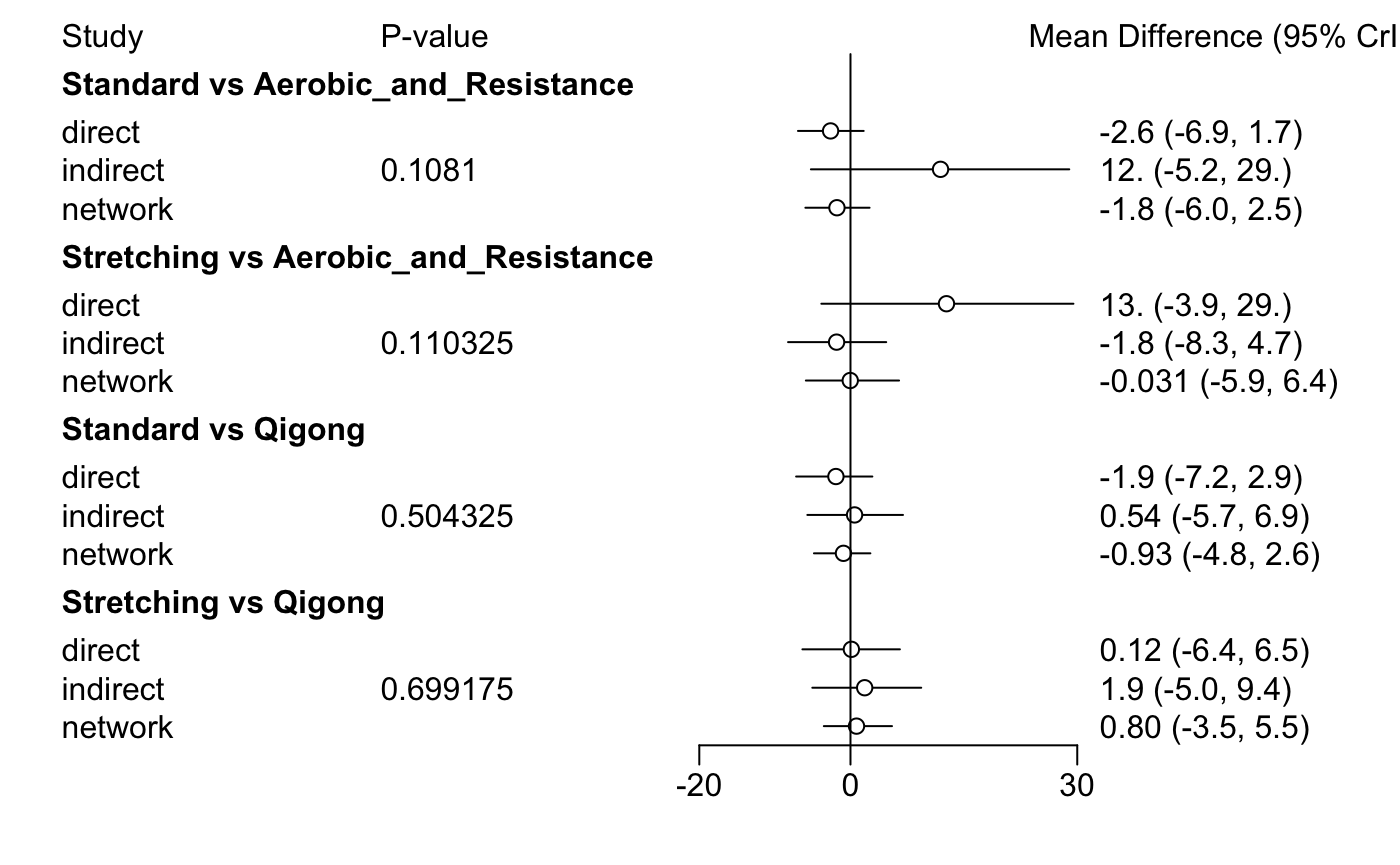
** **
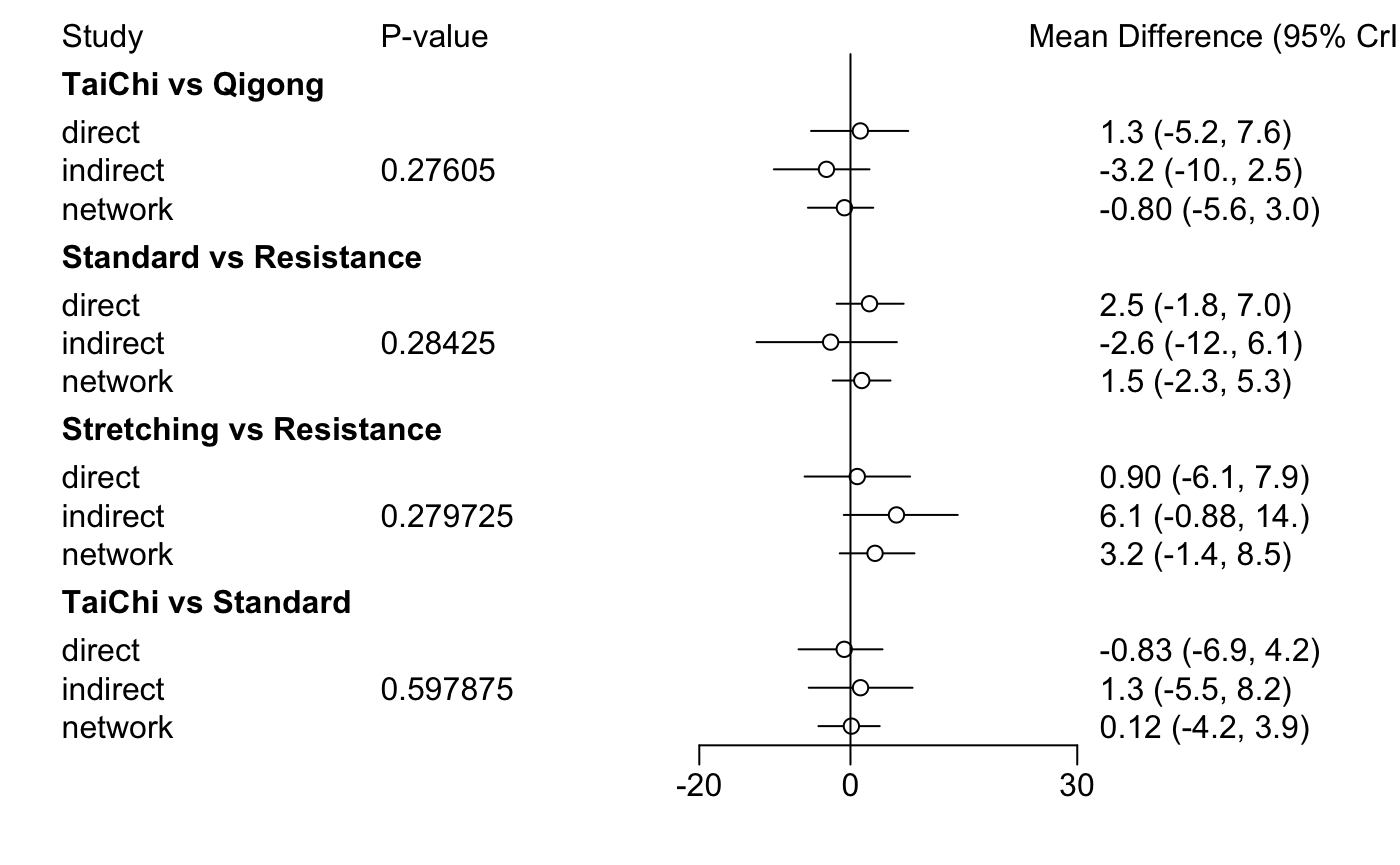
**
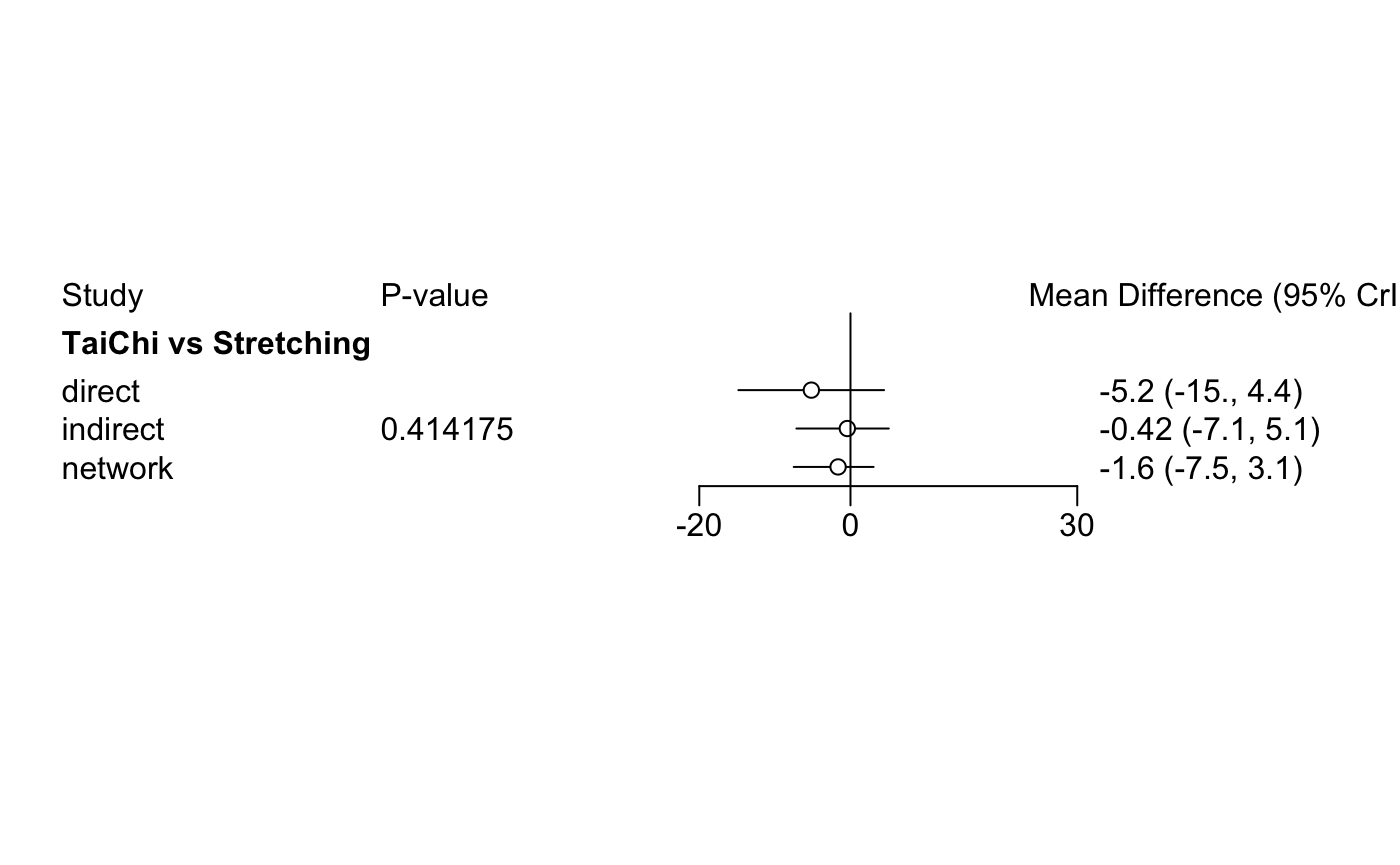


**Figure S3. Sensitivity analysis: excluded reports with a high risk of RoB2**

| Aerobic |  |  |  |  |  |  |
| --- | --- | --- | --- | --- | --- | --- |
| -2.35  (-5.96, 1.35) | Aerobic and Resistance |  |  |  |  |  |
| 4.78  (-2.95, 12.79) | 7.08  (-1.33, 15.24) | Qigong |  |  |  |  |
| 1.67  (-0.18, 3.51) | **4.02**  **(0.44, 7.5)** | -3.10  (-11.08, 4.53) | Resistance |  |  |  |
| 4.63  (-3.10, 12.63) | 6.94  (-1.47, 15.10) | -0.15  (-0.46, 0.16) | 2.95  (-4.70, 10.95) | Stretching |  |  |
| 3.57  (-4.09, 11.56) | 5.86  (-2.52, 13.98) | **-1.22**  **(-2.19, -0.24)** | 1.90  (-5.71, 9.83) | **-1.07**  **(-2.09, -0.05)** | Tai Chi |  |
| **5.57**  **(4.06, 7.09)** | **7.93**  **(4.51, 11.27)** | 0.81  (-7.12, 8.35) | **3.91**  **(2.67, 5.16)** | 0.96  (-6.98, 8.50) | 2.01  (-5.89, 9.52) | Yoga |

**Figure S4. Sensitivity analysis: Forest plot**

**
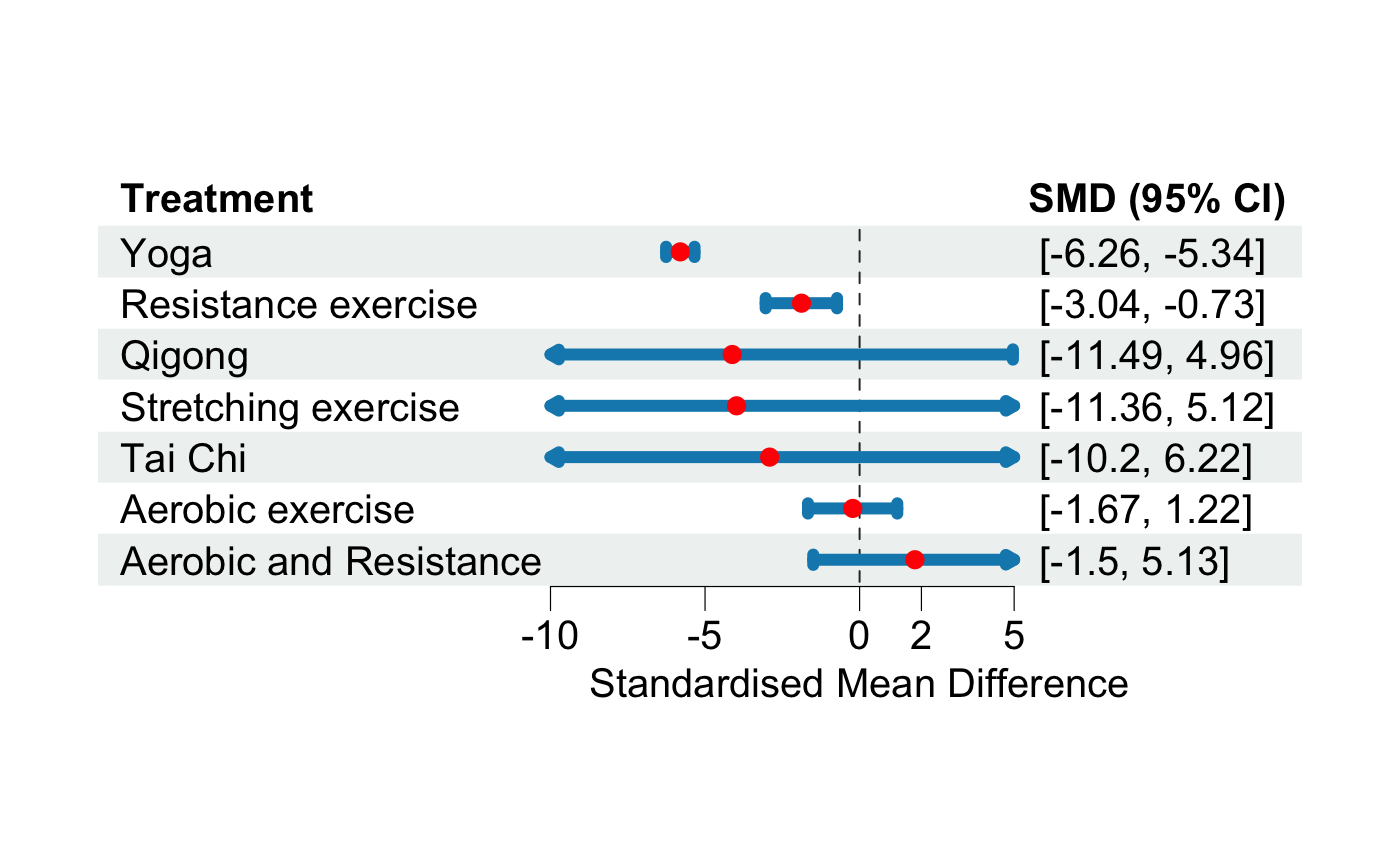
**

**Figure S5. Sensitivity analysis: excluded trials with small sample sizes**

| Aerobic |  |  |  |  |  |  |
| --- | --- | --- | --- | --- | --- | --- |
| -3.26  (-6.96, 0.41) | Aerobic and Resistance |  |  |  |  |  |
| -0.31  (-2.23, 1.60) | 2.95  (-0.49, 6.37) | Qigong |  |  |  |  |
| 3.36  (-0.62, 8.32) | **6.62**  **(0.86, 12.36)** | 3.67  (-1.11, 8.44) | Resistance |  |  |  |
| -0.47  (-2.41, 1.47) | 2.80  (-0.65, 6.24) | -0.15  (-0.46, 0.16) | -3.82  (-8.61, 0,97) | Stretching |  |  |
| -1.58  (-3.48, 0.33) | 1.69  (-1.75, 5.12) | **-1.26**  **(-2.11, -0.42)** | **-4.93**  **(-9.70, -0.15)** | **-1.11**  **(-2.01, -0.22)** | Tai Chi |  |
| 1.23  (-0.44, 2.88) | **4.49**  **(1.19, 7.79)** | **1.54**  **(0.58, 2.50)** | -2.13  (-6.81, 2.56) | **1.70**  **(0.68, 2.70)** | **2.80**  **(1.86, 3.75)** | Yoga |

**Figure S6. Sensitivity analysis: Forest plot**

**
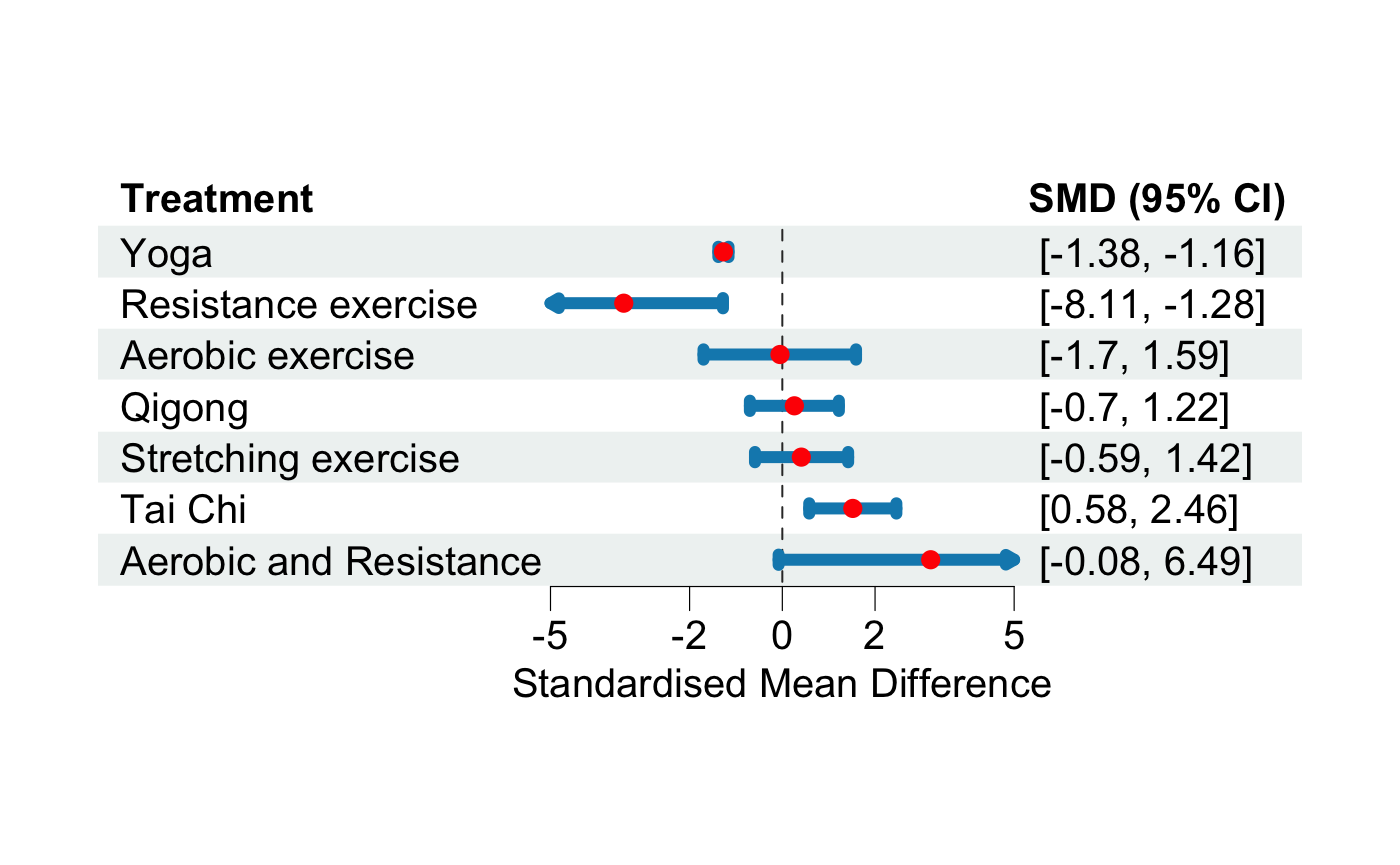
**

**Table S2. GRADE summary of findings table.**

**Question:** Aerobic exercise compared to Mixed exercise in the treatment of cancer-related fatigue

| **Certainty assessment** | | | | | | | **№ of patients** | | **Effect** | **Certainty** | **Importance** |
| --- | --- | --- | --- | --- | --- | --- | --- | --- | --- | --- | --- |
| **№ of studies** | **Study design** | **Risk of bias** | **Inconsistency** | **Indirectness** | **Imprecision** | **Other considerations** | **Aerobic exercise** | **Mixed exercise** | **Relative (95% CI)** |  |  |
| **Fatigue (Outcome): Measured at end of treatment;** **Low score means less fatigue** | | | | | | | | | | | |
| - | Randomized trials | serious | not serious | serious | very serious | none | 281 | 157 | SMD **1.29 SD lower** (7.87 lower to 5.42 higher) | ⨁◯◯◯ Very low | CRITICAL |

**Question:** Qigong compared to Aerobic exercise in the treatment of cancer-related fatigue

| **Certainty assessment** | | | | | | | **№ of patients** | | **Effect** | **Certainty** | **Importance** |
| --- | --- | --- | --- | --- | --- | --- | --- | --- | --- | --- | --- |
| **№ of studies** | **Study design** | **Risk of bias** | **Inconsistency** | **Indirectness** | **Imprecision** | **Other considerations** | **Qigong** | **Aerobic exercise** | **Relative (95% CI)** |  |  |
| **Fatigue (Outcome): Measured at end of treatment; Low score means less fatigue** | | | | | | | | | | | |
| - | Randomized trials | serious | serious | not serious | very serious | none | 191 | 281 | SMD **3.52 SD lower** (11.52 lower to 4.43 higher) | ⨁◯◯◯ Very low | CRITICAL |

**Question:** Qigong compared to Mixed exercise in the treatment of cancer-related fatigue

**Question:** Qigong compared to Mixed exercise in the treatment of cancer-related fatigue

| **Certainty assessment** | | | | | | | **№ of patients** | | **Effect** | **Certainty** | **Importance** |
| --- | --- | --- | --- | --- | --- | --- | --- | --- | --- | --- | --- |
| **№ of studies** | **Study design** | **Risk of bias** | **Inconsistency** | **Indirectness** | **Imprecision** | **Other considerations** | **Qigong** | **Mixed exercise** | **Relative (95% CI)** |  |  |
| **Fatigue (Outcome): Measured at end of treatment; Low score means less fatigue** | | | | | | | | | | | |
| - | Randomized trials | serious | not serious | not serious | very serious | none | 191 | 157 | SMD **2.24 SD lower** (10.7 lower to 6.09 higher) | ⨁◯◯◯ Very low | CRITICAL |

**Question:** Resistance exercise compared to Aerobic exercise in the treatment of cancer-related fatigue

| **Certainty assessment** | | | | | | | **№ of patients** | | **Effect** | **Certainty** | **Importance** |
| --- | --- | --- | --- | --- | --- | --- | --- | --- | --- | --- | --- |
| **№ of studies** | **Study design** | **Risk of bias** | **Inconsistency** | **Indirectness** | **Imprecision** | **Other considerations** | **Resistance exercise** | **Aerobic exercise** | **Relative (95% CI)** |  |  |
| **Fatigue (Outcome): Measured at end of treatment; Low score means less fatigue** | | | | | | | | | | | |
| - | Randomized trials | not serious | not serious | serious | very serious | none | 136 | 281 | SMD **0.69 SD lower** (7.94 lower to 6.45 higher) | ⨁◯◯◯ Very low | CRITICAL |

**Question:** Resistance exercise compared to Mixed exercise in the treatment of cancer-related fatigue

| **Certainty assessment** | | | | | | | **№ of patients** | | **Effect** | **Certainty** | **Importance** |
| --- | --- | --- | --- | --- | --- | --- | --- | --- | --- | --- | --- |
| **№ of studies** | **Study design** | **Risk of bias** | **Inconsistency** | **Indirectness** | **Imprecision** | **Other considerations** | **Resistance exercise** | **Mixed exercise** | **Relative (95% CI)** |  |  |
| **Fatigue (Outcome): Measured at end of treatment; Low score means less fatigue** | | | | | | | | | | | |
| - | Randomized trials | not serious | not serious | serious | very serious | none | 136 | 157 | SMD **0.6 SD higher** (7.23 lower to 8.24 higher) | ⨁◯◯◯ Very low | CRITICAL |

**Question:** Resistance exercise compared to Qigong in the treatment of cancer-related fatigue

| **Certainty assessment** | | | | | | | **№ of patients** | | **Effect** | **Certainty** | **Importance** |
| --- | --- | --- | --- | --- | --- | --- | --- | --- | --- | --- | --- |
| **№ of studies** | **Study design** | **Risk of bias** | **Inconsistency** | **Indirectness** | **Imprecision** | **Other considerations** | **Resistance exercise** | **Qigong** | **Relative (95% CI)** |  |  |
| **Fatigue (Outcome): Measured at end of treatment; Low score means less fatigue** | | | | | | | | | | | |
| - | Randomized trials | not serious | not serious | serious | very serious | none | 136 | 191 | SMD **2.84 SD higher** (5.65 lower to 11.3 higher) | ⨁◯◯◯ Very low | CRITICAL |

**Question:** Tai Chi compared to Aerobic exercise in the treatment of cancer-related fatigue

| **Certainty assessment** | | | | | | | **№ of patients** | | **Effect** | **Certainty** | **Importance** |
| --- | --- | --- | --- | --- | --- | --- | --- | --- | --- | --- | --- |
| **№ of studies** | **Study design** | **Risk of bias** | **Inconsistency** | **Indirectness** | **Imprecision** | **Other considerations** | **Tai Chi** | **Aerobic exercise** | **Absolute (95% CI)** |  |  |
| **Fatigue (Outcome): Measured at end of treatment; Low score means less fatigue** | | | | | | | | | | | |
| - | Randomized trials | not serious | not serious | serious | very serious | none | 150 | 281 | SMD **0.56 SD lower** (8.44 lower to 7.27 higher) | ⨁◯◯◯ Very low | CRITICAL |

**Question:** Tai Chi compared to Mixed exercise in the treatment of cancer-related fatigue

| **Certainty assessment** | | | | | | | **№ of patients** | | **Effect** | **Certainty** | **Importance** |
| --- | --- | --- | --- | --- | --- | --- | --- | --- | --- | --- | --- |
| **№ of studies** | **Study design** | **Risk of bias** | **Inconsistency** | **Indirectness** | **Imprecision** | **Other considerations** | **Tai Chi** | **Mixed exercise** | **Relative (95% CI)** |  |  |
| **Fatigue (Outcome): Measured at end of treatment; Low score means less fatigue** | | | | | | | | | | | |
| - | Randomized trials | not serious | serious | serious | very serious | none | 150 | 157 | SMD **0.73 SD higher** (7.52 lower to 8.85 higher) | ⨁◯◯◯ Very low | CRITICAL |

**Question:** Tai Chi compared to Qigong in the treatment of cancer-related fatigue

| **Certainty assessment** | | | | | | | **№ of patients** | | **Effect** | **Certainty** | **Importance** |
| --- | --- | --- | --- | --- | --- | --- | --- | --- | --- | --- | --- |
| **№ of studies** | **Study design** | **Risk of bias** | **Inconsistency** | **Indirectness** | **Imprecision** | **Other considerations** | **Tai Chi** | **Qigong** | **Relative (95% CI)** |  |  |
| **Fatigue (Outcome): Measured at end of treatment; Low score means less fatigue** | | | | | | | | | | | |
| 1 | Randomized trials | not serious | not serious | serious | not serious | none | 150 | 191 | -SMD **2.98 SD higher** (4.82 lower to 10.75 higher) | ⨁⨁⨁◯ Moderate | CRITICAL |

**Question:** Tai Chi compared to Resistance exercise in the treatment of cancer-related fatigue

| **Certainty assessment** | | | | | | | **№ of patients** | | **Effect** | **Certainty** | **Importance** |
| --- | --- | --- | --- | --- | --- | --- | --- | --- | --- | --- | --- |
| **№ of studies** | **Study design** | **Risk of bias** | **Inconsistency** | **Indirectness** | **Imprecision** | **Other considerations** | **Tai Chi** | **Resistance exercise** | **Relative (95% CI)** |  |  |
| **Fatigue (Outcome): Measured at end of treatment; Low score means less fatigue** | | | | | | | | | | | |
| - | Randomized trials | not serious | not serious | serious | very serious | none | 150 | 136 | SMD **0.13 SD higher** (8.31 lower to 8.68 higher) | ⨁◯◯◯ Very low | CRITICAL |

**Question:** Yoga compared to Aerobic exercise in the treatment of cancer-related fatigue

| **Certainty assessment** | | | | | | | **№ of patients** | | **Effect** | **Certainty** | **Importance** |
| --- | --- | --- | --- | --- | --- | --- | --- | --- | --- | --- | --- |
| **№ of studies** | **Study design** | **Risk of bias** | **Inconsistency** | **Indirectness** | **Imprecision** | **Other considerations** | **Yoga** | **Aerobic exercise** | **Relative (95% CI)** |  |  |
| **Fatigue (Outcome): Measured at end of treatment; Low score means less fatigue** | | | | | | | | | | | |
| - | Randomized trials | not serious | not serious | not serious | very serious | none | 436 | 281 | SMD **2.8 SD higher** (3.33 lower to 8.97 higher) | ⨁⨁◯◯ Low | CRITICAL |

**Question:** Yoga compared to Mixed exercise in the treatment of cancer-related fatigue

| **Certainty assessment** | | | | | | | **№ of patients** | | **Effect** | **Certainty** | **Importance** |
| --- | --- | --- | --- | --- | --- | --- | --- | --- | --- | --- | --- |
| **№ of studies** | **Study design** | **Risk of bias** | **Inconsistency** | **Indirectness** | **Imprecision** | **Other considerations** | **Yoga** | **Mixed exercise** | **Relative (95% CI)** |  |  |
| **Fatigue (Outcome): Measured at end of treatment; Low score means less fatigue** | | | | | | | | | | | |
| - | Randomized trials | not serious | serious | serious | very serious | none | 436 | 157 | SMD **4.08 SD higher** (2.9 lower to 11.01 higher) | ⨁◯◯◯ Very low | CRITICAL |

**Question:** Yoga compared to Qigong in the treatment of cancer-related fatigue

| **Certainty assessment** | | | | | | | **№ of patients** | | **Effect** | **Certainty** | **Importance** |
| --- | --- | --- | --- | --- | --- | --- | --- | --- | --- | --- | --- |
| **№ of studies** | **Study design** | **Risk of bias** | **Inconsistency** | **Indirectness** | **Imprecision** | **Other considerations** | **Yoga** | **Qigong** | **Relative (95% CI)** |  |  |
| **Fatigue (Outcome): Measured at end of treatment; Low score means less fatigue** | | | | | | | | | | | |
| - | Randomized trials | not serious | not serious | serious | very serious | none | 436 | 191 | SMD **6.33 SD higher** (1.08 lower to 14.54 higher) | ⨁◯◯◯ Very low | CRITICAL |

**Question:** Yoga compared to Resistance exercise in the treatment of cancer-related fatigue

| **Certainty assessment** | | | | | | | **№ of patients** | | **Effect** | **Certainty** | **Importance** |
| --- | --- | --- | --- | --- | --- | --- | --- | --- | --- | --- | --- |
| **№ of studies** | **Study design** | **Risk of bias** | **Inconsistency** | **Indirectness** | **Imprecision** | **Other considerations** | **Yoga** | **Resistance exercise** | **Relative (95% CI)** |  |  |
| **Fatigue (Outcome): Measured at end of treatment; Low score means less fatigue** | | | | | | | | | | | |
| - | Randomized trials | not serious | not serious | serious | very serious | none | 436 | 136 | SMD **3.48 SD higher** (3.89 lower to 11.03 higher) | ⨁◯◯◯ Very low | CRITICAL |

**Question:** Yoga compared to Tai Chi in the treatment of cancer-related fatigue

| **Certainty assessment** | | | | | | | **№ of patients** | | **Effect** | **Certainty** | **Importance** |
| --- | --- | --- | --- | --- | --- | --- | --- | --- | --- | --- | --- |
| **№ of studies** | **Study design** | **Risk of bias** | **Inconsistency** | **Indirectness** | **Imprecision** | **Other considerations** | **Yoga** | **Tai Chi** | **Relative (95% CI)** |  |  |
| **Fatigue (Outcome): Measured at end of treatment; Low score means less fatigue** | | | | | | | | | | | |
| - | Randomized trials | not serious | not serious | serious | very serious | none | 436 | 150 | SMD **3.36 SD higher** (4.73 lower to 11.5 higher) | ⨁◯◯◯ Very low | CRITICAL |

**CI:** confidence interval; **SMD:** standardized mean difference
